# Genome-Wide Detection of SNP and SV Variations to Reveal Early Ripening-Related Genes in Grape

**DOI:** 10.1371/journal.pone.0147749

**Published:** 2016-02-03

**Authors:** Yanshuai Xu, Zhihong Gao, Jianmin Tao, Weihua Jiang, Shijie Zhang, Qiunan Wang, Shenchun Qu

**Affiliations:** 1 College of Horticulture, Nanjing Agricultural University, No. 1 Weigang, Nanjing, 210095, P. R. China; 2 Agricultural Bureau & Forestry Workstation, Wujin District, Changzhou, 213000, P. R. China; 3 Jiangsu Key Laboratory for Horticultural Crop Genetic Improvement, No.50 Zhongling Street, Nanjing, 210014, P.R. China; Key Laboratory of Horticultural Plant Biology (MOE), CHINA

## Abstract

Early ripening in grape (*Vitis vinifera* L.) is a crucial agronomic trait. The fruits of the grape line ‘Summer Black’ (SBBM), which contains a bud mutation, can be harvested approximately one week earlier than the ‘Summer Black’ (SBC)control. To investigate the molecular mechanism of the bud mutation related to early ripening, we detected genome-wide genetic variations based on re-sequencing. In total, 3,692,777 single nucleotide polymorphisms (SNPs) and 81,223 structure variations (SVs) in the SBC genome and 3,823,464 SNPs and 85,801 SVs in the SBBM genome were detected compared with the reference grape sequence. Of these, 635 SBC-specific genes and 665 SBBM-specific genes were screened. Ripening and colour-associated unigenes with non-synonymous mutations (NS), SVs or frame-shift mutations (F) were analysed. The results showed that 90 unigenes in SBC, 76 unigenes in SBBM and 13 genes that mapped to large fragment indels were filtered. The expression patterns of eight genes were confirmed using quantitative reverse transcription-polymerase chain reaction (qRT-PCR).The re-sequencing data showed that 635 SBC-specific genes and 665 SBBM-specific genes associated with early ripening were screened. Among these, *NCED6* expression appears to be related to *NCED1* and is involved in ABA biosynthesis in grape, which might play a role in the onset of anthocyanin accumulation. The *SEP* and *ERF* genes probably play roles in ethylene response.

## Introduction

Grapevine (*Vitis vinifera* L.) is one of most important fruit crops worldwide, as it provides fruit used for both table grapes and wine. The genome size of grapevine is about 475–500 Mb [1] and consists of 19 chromosomes (chr) with a high degree of heterozygosity [2]. Early ripening is an attractive trait for producers, especially in areas when heavy rain occurs at the time of the year when the fruits are harvested. However, the mechanism of fruit ripening in grape is not clear.

Fruit ripening is affected by many factors, such as natural environmental conditions, including temperature, water, sunlight, diseases, turgor, and sugar accumulation [3]. Furthermore, plant hormones, especially ethylene (ETH) and abscisicacid (ABA) play a decisive role in regulating the growth and development of fruits [4, 5]. Plant hormones such as ETH and ABA promote ripening, whereas auxins (IAAs) inhibit berry ripening and colouring in grape [6], and the role of cytokinins (CTKs) is unclear. Böttcher et al. found that the CTK level increased during the ripening of *Vitis vinifera cv*. Shiraz berries [7]. On the basis of the fairly stable and low respiration rates and the low levels of ethylene in berries, grape has been defined as a non-climacteric fruit [8]. Previous research suggested that the plant hormone ABA might be involved in the regulation of non-climacteric berry ripening [9, 10], whereas the function of ETH in the ripening of grape was not very clear. Brassinosteroids (BRs) are also involved in strawberry fruit ripening [11]. In addition, anthocyanins affect the appearance of fruit, and an increasing concentrate of anthocyanin contributes to fruit maturation [12, 13]. Other significant pathways, such as the accumulation of sugar, berry softening, the catabolism of organic acids, and flavour maturation at the onset of véraison in grape are associated with fruit ripening [14]. Sequence variation and structural alteration belong to genetic variation. Normally, sequence variation involves SNPs, indels, transposable elements and microsatellites, similar to the humansickle-cell anaemia and cystic fibrosis diseases, which are caused by one or three nucleotide changes in the haemoglobin beta gene, respectively [15]. Structural variation is generally described as large scale deletions, insertions, duplications, inversions and translocations. Whole genome amplicon re-sequencing has provided much information on genetic variation and gene function in biological and medical studies, as well as in the validation and assessment of SNPs and SVs involving indels and copy number variants (CNVs) in two grape lines by comparison with the reference genome. Furthermore, robust next generation sequencing (NGS) platforms for *in silico* SNP identification have supplied a very effective alternative to re-sequencing for SNPs studies in plants [16]. Abundant SNPs and indel markers have become a powerful tool for many applications, including marker assisted selection, association studies, diversity analysis, genetic mapping, and the map-based cloning of genes [17–19].

The ‘Summer Black’ grape is a seedless, triploid grape cultivar with a dark, purple-black pericarp, it is a European and American hybrid, derived from the seedless grape cultivars ‘Kyoho’ and ‘Thompson’ [20]. ‘Summer Black’ possesses many advantageous horticultural traits, including a strong resistance, high yield, very early maturation, easy colouring, strong growth, and the ability to withstand storage and transportation. The bud germination rate is 85%–90%, the graft rate is 95%, and the yield is 30,600–37,500 kg•hectare^-1^[21] and it can be planted throughout China. We have found a precocious bud mutation in the grapevines of the ‘Summer Black’ line (SBBM). Since the fruits mature a week earlier, the SBBM cultivar might potentially be economically valuable. Given the importance of this mutation, whole genome re-sequencing was performed and simple sequence repeat (SSR) molecular markers were used, in addition to measurements of the content of hormones and anthocyanins, to assess the differences between SBBM and control ‘Summer Black’ (SBC) at the molecular and physiological levels.

## Methods

### Ethical Statement

This study was carried out in a private vineyard, and permission of the vineyard owner was obtained. The plant material used was a common grapevine variety and was not endangered.

### Treatment of plant materials

**Fig 1:**
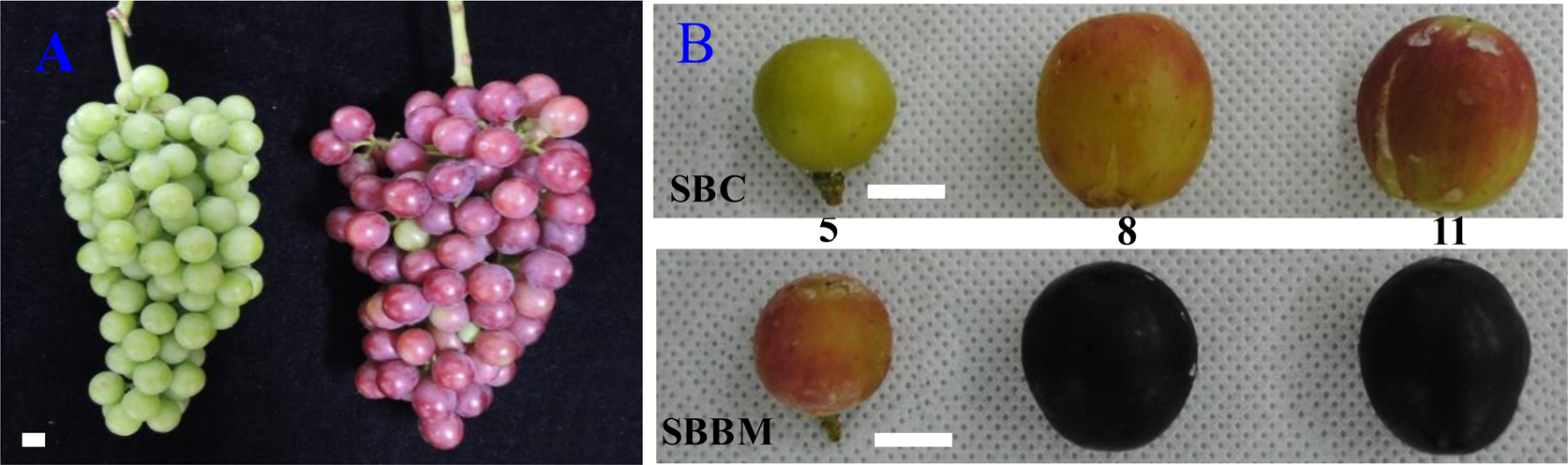
The grapes materials used in this study.

The two grapevines (SBC and SBBM) were planted under natural illumination in Changzhou City, Jiangsu Province, China. By observing the phenotypic agronomic traits, we found that SBBM colouring and ripening occurred approximately one week earlier than in SBC. We harvested young leaves of the two cultivated grape lines to perform genome re-sequencing, and determined the DNA level of SNPs and SVs, and assessed differences in gene expression. The young leaves were stored at -70°Cimmediately, and were used to extract DNA. The grapes used to determine the hormone content were harvested on 17 June, 8 July, and 29 July. The ‘Summer Black’ grapes were harvested five, eight and eleven weeks after flowering, respectively (Fig 1B).

### Investigation of physiological indicators and agricultural traits

**Fig 2:**
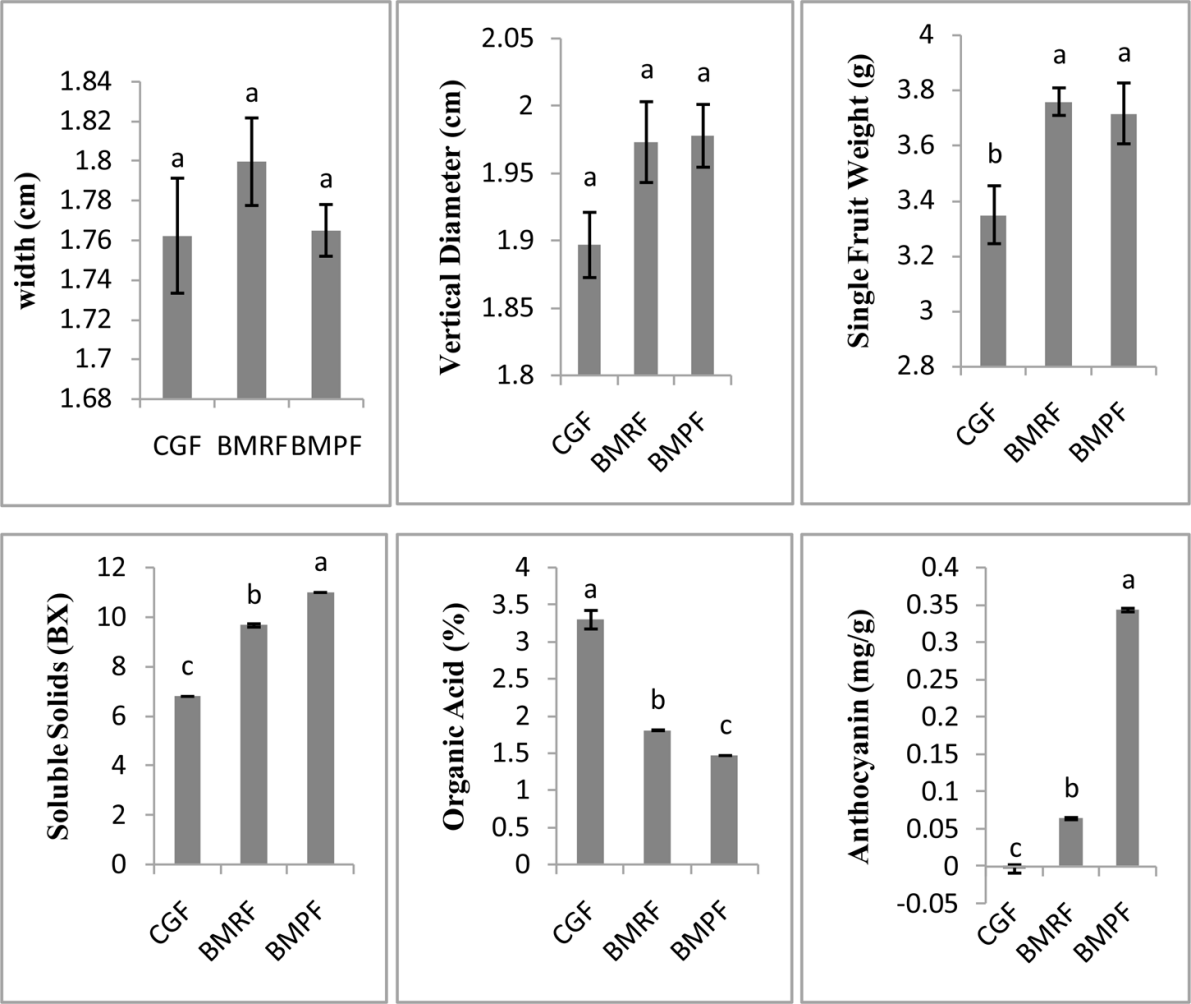
The differences of physiological indicators at five weeks after flowering.

The control ‘Summer Black’ grape lines are represented by CGF, BMRF represents the red grapes of the bud mutation line and BMPF represents the purple grapes of the bud mutation line (Fig 1A and Fig 2). Physiological indicators were measured at the three different grape ripeness time points in Fig 1A. The vertical diameter and width of grapes were measured using a Verniercaliper. The content of soluble solids was determined using a hand saccharimeter, the cover plate was opened and a drop of juice from a crushed grape was placed on the cleaned detection prism. Three replicate measurements were performed.

The organic acid content was measured using an acid–base titration method with some modifications. Firstly, 30 skinned grapes were squeezed to obtain juice, which was then centrifuged at 4°C and 8,000 rpm for 30 min and incubated at 75–80°C in a water bath for 30 min. Subsequently, 1 mL of the supernatant was added to 30 mL distilled water and two drops of 1% phenolphthalein in a 150mL flask. Then, using NaOH, the solution was titrated until the pink colour remained for 30 s without fading, and the volume of NaOH added was recorded for three replicates. The results were calculated using the following formula: Total acidity (%) = V_2_×N×conversion coefficient×100/V_1_, where V_1_ represents the volume of the sample titration solution (mL), V_2_ represents the volume of the standard solution of sodium hydroxide consumed (mL), and N represents the molar concentration of the sodium hydroxide standard solution (mol/L), with a conversion coefficient for tartaric acid of 0.075 [22].

For the anthocyanin assay, two grape lines were ground in liquid nitrogen (by adding 1 g of grape powder to achieve mixing uniformity) and the powder was resuspended in 10 mL 1% concentrated HCl at room temperature. Ultrasonic vibration was then provided for 30 min at 100 W and 40 KHz. The brei was centrifuged for 30 min in glass tubes at 8,000 rpm and 4°C and the supernatant was decanted and another 10 mL of the solvent was added to the residue. The brei was placed in the dark, the process was repeated, and the supernatant was diluted to a final volume of 25 mL. Next, 6 mL of the supernatant and 9 mL KCl/HCl buffersolution (pH 1.0) were incubated for 50 min and 6 mL of the supernatant and 9 mL citric acid/disodium hydrogen phosphate buffersolution (pH 5.0) were incubated for 80 min in the dark. The total anthocyanin content was then measured using the mixed solvent (5 mL) by determining the absorbance at 510 mm using a Pye Unicam recording spectrophotometer. Three replicate measurements were performed [23].

The concentration of hormones was determined using an enzyme-linked immunosorbent assay (icELISA) [24]. The grape samples were ground and centrifuged at 3,500 rpm for 8 min after 4 hours at 4°C. The supernatant was filtered through a C-18 solid phase extraction column. The results were calculated using the logit curve as follows: Logit (B/B0) = ln (B/B0)/(1-B/B0) = ln B/(B0-B), where B_0_ is the chromogenic value at 0 ng/mL and B is the chromogenic value of the other samples. The logarithm value corresponds to the value in the logit curve. The hormone content (ng/mL) was obtained by calculating the antilog value (ng/mL). All the above data were analysed by ANOVA using IBM SPSS Statistics 19.

### Genomic variation identified by SSR

The DNA of the two grape lines was extracted using the CTAB method[25]. The concentration of each DNA sample was measured using a spectrophotometer at 260 and 280 nm and each DNA sample was also quantified on an agarose gel. The 48 pairs of SSR primers were designed using Primer Premier 5 software and PCR was performed in a final volume of 20 μL. The PCR mixture was as follows: 1.5 μL (100 ng/μL) DNA template, 1 μL each primer, 2 μL 10× PCR reaction buffer (without MgCl_2_), 1.6 mM dNTP mix, 1.2 μL MgCl_2_, 0.2 μL *rTaq* DNA polymerase and 11.5 μL ddH_2_O. The PCR was carried out in a thermocycler with the following protocol: 94°C for 5 min, 35 cycles with 94°C denaturation for 50 s, annealing for 55 s, and 73°C elongation for 1 min, followed by 4°C elongation for 10 min. All PCR products were validated by analysis on a polyacrylamide gel.

### DNA variation identified by whole-genome re-sequencing

Whole-genome re-sequencing of the grape lines was performed on an Illumina HiSeq2000™ by Biomarker Technologies (Beijing, China). The procedure was performed in accordance with the standard Illumina protocol, including sample preparation and sequencing as follows: quantified DNA was extracted and DNA fragments were obtained using ultrasound, which was purified using the QIA quick PCR kit. End repair was performed with poly-A on the 3´ ends, then the adaptors were ligated, clusters were generated, agarose gel electrophoresis was used to select fragments, and PCR amplification was performed. Sequencing was performed by establishing a library with Illumina HiSeq2000™. The short reads were aligned using the Burrows–Wheeler transformation [26].

### SNP and SV screening

According to the results of our data and comparison with the reference genome sequence, we used SAMtools [27] software (http://samtools.souRceforge.net/Samtools.shtml) to detect SNPs by filtering the bases of low depth and low quality (minimum depth of 2×, and that for a heterozygous SNP is 3×, the maximum depth did not exceed 3× of the average depth, and the genotype quality value was not less than 20 and was not located in simple sequence repeat regions). We obtained a high reliability mismatch (mismatch of base) loci array, which indicated single molecular polymorphism (SNP) sites.

Pindel [28] and BreakDancer [29] software was applied for SNP and SV detection. Based on BAM and SAM (short DNA sequence read alignments in the *SAM*, *BAM* formats) data types, Pindel (https://trac.nbic.nl/pindel/) is a software package designed specifically for bwa (Public Library of Bioinformatics) software to detect structural variations in genes. BreakDancer (http://breakdancer.sourceforge.net/breakdancermax.html) can predict six types of SV sites, including INS (insertions), DEL (deletions), IDE (insertion inside deletions), INV (inversions), ITX (intra-chromosome transfers), and CTX (inter-chromosome transfers).

### DNA level functional annotation categories

The mainly functional annotation of genes compared the differences in gene expression with various annotation databases, in which unigene annotation information was represented by annotated homologous genes. The NR, SwissProt, GO, COG, and KEGG annotation databases were used to compare homologous genes in our approach. The five linkage websites are listed in S1 Table.

### The expression pattern of genes related to ripening and colour assessed by qRT-PCR

Expression differences in ripening and colour relevant genes were validated using qRT-PCR. Total RNA was extracted using the CTAB method. Agarose gel electrophoresis was used to test the products, and the concentration of total RNA was measured using a micro-spectrophotometer. Reverse transcription of total RNA into cDNA was performed using a transcription kit. In the next step of the qRT-PCR experiment, the cDNA was diluted to100 ng/μL. The specific primers were designed using Beacon designer software. The 18S gene was selected as the housekeeping gene. The qRT-PCR was performed in 96well plates with a total volume of 20 μL: 10 μL SYBR Green II Master, 8.6 μL ddH_2_O, 1 μL (100 ng/μL) of the cDNA template, and 0.2 μL each primer. The reaction was performed on a 7300 Real-Time PCR System (Applied Biosystems, Foster City, CA). All genes were assessed in triplicate and the reactions used for qRT-PCR are listed in Table 1. Gene expression is presented as relative expression levels calculated using the 2^-ΔΔXT^ method.

**Table 1:** Primers pairs used for qRT-PCR.

Gene	Gene ID	Forward primer	Reverse primer
*CHSY*	GSVIVT01032968001	5´-ATGGTGGTGGTTGAAGTA-3´	5´-GCTTGGTGAGTTGATAGTC-3´
*3MAT*	GSVIVT01000461001	5´-GGTGAGGATGTTGGTGAA-3´	5´-GACTTGGCTGCGACTATA-3
*ERF5*	GSVIVT01013934001	5´-ACCTTCCAGATTGAGTCAT-3´	5´-CCTAGATCCTCTCCGATTC-3´
*ERF03*	GSVIVT01026334001	5´-ACCTTCCAGATTGAGTCAT-3´	5´-CCTAGATCCTCTCCGATTC-3´
*AI5L2*	GSVIVT01033216001	5´-GCCTTATCTTCTCCATTGATG-3	5´-TGTTCTCCAGTTCATTTGTG-3
*SEP1*	GSVIVT01008139001	5´-TGGGAGATTGATGGGATTT-3	5´-GGCTACAAGGTTTGGAAAG-3´
*PT106*	GSVIVT01020780001	5´-TATGCTTGCTGCTGTTATG-3´	5´-GCTCATCAATGTAAGTGGA-3´
*NCED6*	GSVIVT01029057001	5´-CATATACCTGGCGATAGCA-3´	5´-CTTCTCCTTCTCCTCCTTC-3´

Note: Gene function annotation. *CHSY*: Chalcone synthase. *3MAT*: Malonyl-coenzyme, A: anthocyanin3-O-glucoside-6-O-malonyltransferase. *ERF5*:Ethylene-responsive transcription factor. *ERF03*: Ethylene-responsive transcription factor. *AI5L2*: ABSCISIC ACID-INSENSITIVE 5-like protein. *SEP1*: Developmental protein SEPALLATA1. *PT106*: Probable sugar phosphate/phosphate translocator. *NCED6*:9-cis-epoxycarotenoid dioxygenase NCED6, chloroplastic.

## Results

### Variation in phenotypic agronomic traits and plant hormones between SBBM and SBC

Phenotypic observation showed that the main difference between SBBM and SBC was the ripening date, with SBBM ripening approximately one week earlier than SBC (Fig 1). Physiological indicators showed no differences in the vertical diameter or cross diameter, which were about 1.8 cm and 1.9 cm, respectively. The SBBM line had a higher single fruit weight than SBC, of about 3.7g for SBBM and 3.3g for SBC. When the pericarp colour deepened, the content of soluble solids increased, and was 6.8 brix (BX) for CGF, 9.7 BX for BMRF and 11 BX for BMPF, and the content of organic acid decreased and was 3.3% for CGF, 1.81%for BMRF and 1.47% for BMPF. The content of anthocyanin increased: -0.003 mg/g for CGF, 0.643 mg/gfor BMRF and 0.343 mg/g for BMPF (Fig 2).

**Fig 3:**
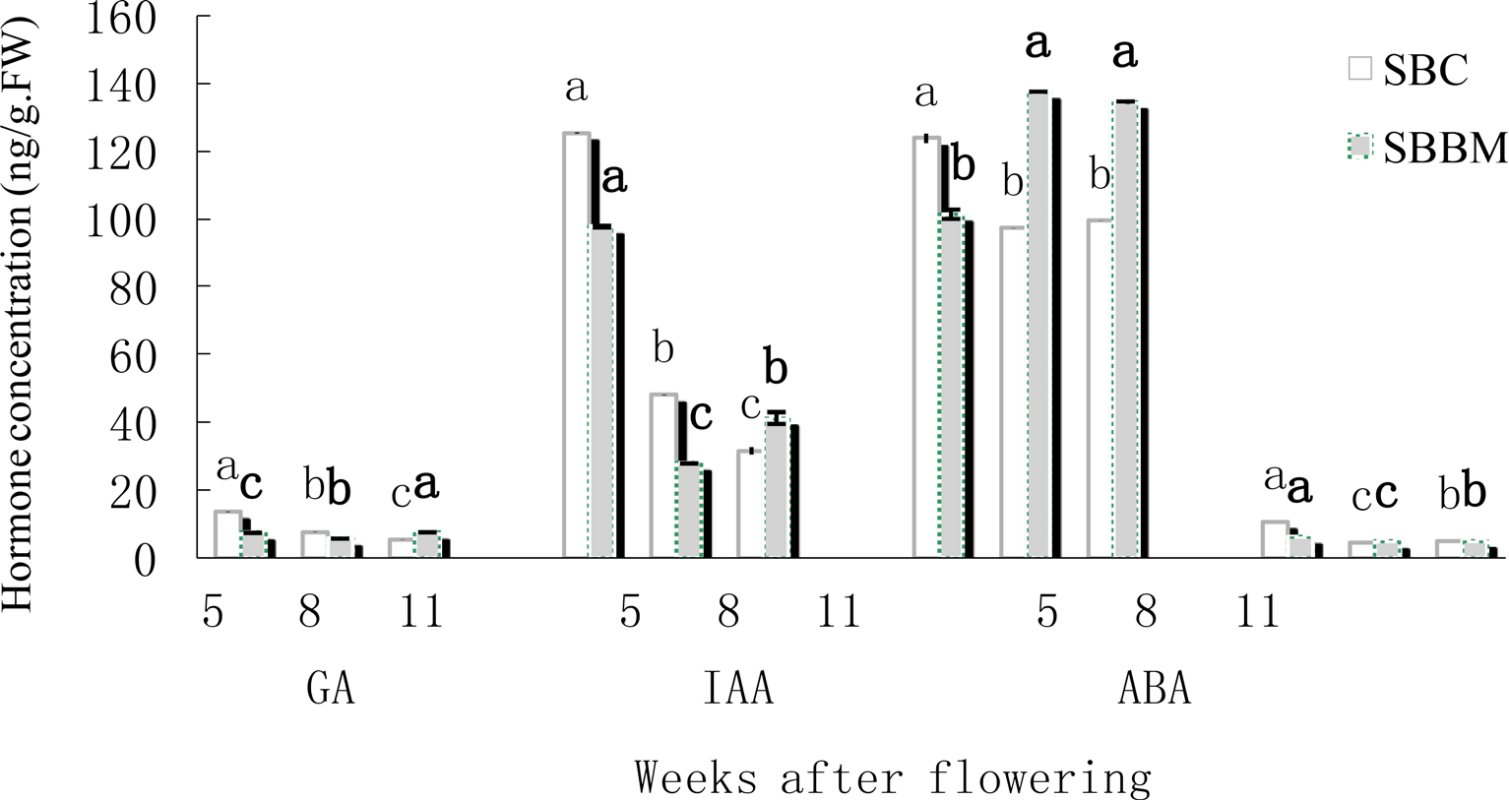
The concentration of GA, IAA, ABA, BR at five, eight and 11 weeks after flowering.

Because hormones regulate plant developmental processes, we measured the content of gibberellin (GA), auxin (IAA),abscisic acid (ABA) and brassinosteroid (BR) by enzyme-linked immunosorbent assays (icELISA). At the three different timepoints, the content of GA in SBC gradually decreased, whereas the concentration of GA in SBBM increased, but the content was always very low, i.e., up to 20 ng•g^-1^ FW (fresh weight) during the ripening process (Fig 3). The IAA level in SBC decreased gradually, and the concentration of IAA in SBBM was overall lower, but there was no obvious regular pattern in SBBM.

Abscisic acid plays a vital role in the maturation of grape berries. As expected, our results also showed that the endogenous ABA concentration in SBC berries decreased from the onset of ripening, but increased in SBBM from véraison to maturation. The concentration of ABA was nearly 140 ng•g^-1^ FW, which was the highest concentration among the four hormones. The concentration of BR in SBC and SBBM decreased and then increased. The concentration of BR was lowest compared with that of GA, IAA and ABA, i.e., less than 15 ng•g^-1^ FW.

### SSR molecular markers

**Fig 4:**
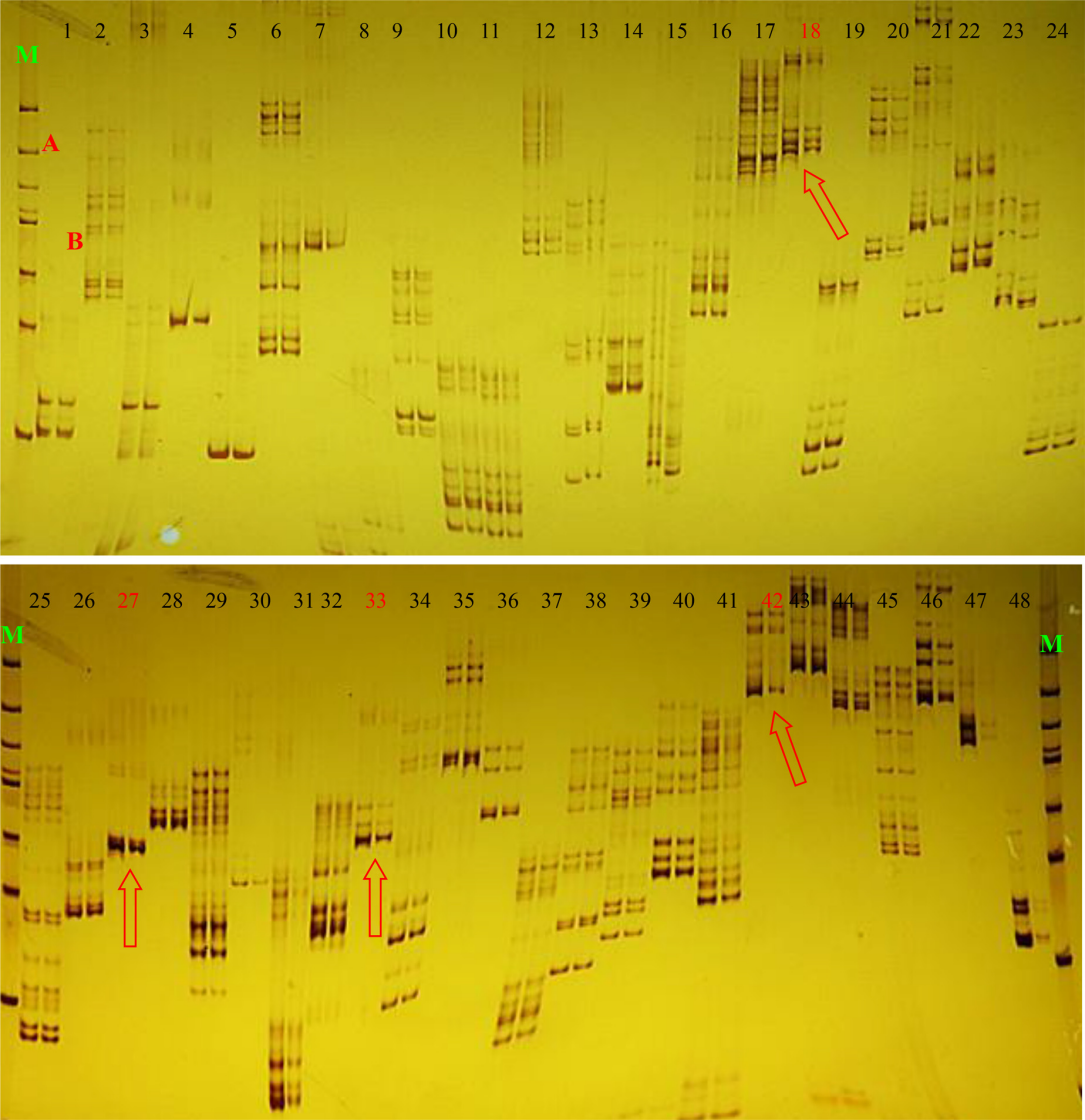
48 pairs of SSR were used to verify the difference between ‘SBC’ and ‘SBBM’.

We investigated SSR molecular markers to analyse the diversity of SBBM and SBC. After polyacrylamide gel electrophoresis, bands were visualised using silver staining. Multiple band patterns were produced and the clearest polymorphisms were detected in each sample. Forty-eight pairs of SSR primers (S2 Table) yielded on average one to eleven bands; three primers yielded the minimum number of bands (1.00). The results reveal that the most reliable polymorphic bands were the same in the two samples, whereas primer 18 (F: 5´-ATTAACGAGGATGTGTTTGG-3´. R: 5´-AAGGATCCATTTCACATACG-3´), primer 27 (F: 5´-ATCTGACAAAGGAAAGGAGAA-3´, R:5´-GTAACATACCGAGGAGGCA-3´), primer 33 (F: 5´-GGTACATCAGTACTTGAAATGGTTGC-3´, R:5´-TTCTCCGTAGAAGCGTAAACAGC-3´), primer 42 (F: 5´-TTATCTGCTTAGGGAAAACGTA-3´, R:5´-AACACACCTTGAGAAAATAGCA-3´) could be clearly distinguished (Fig 4). Compared with SBBM, a band appeared for SBC at the downmost bands which were amplified by primers 18, 27, 33 and 42.

### SNP and SV catalogue

**Fig 5:**
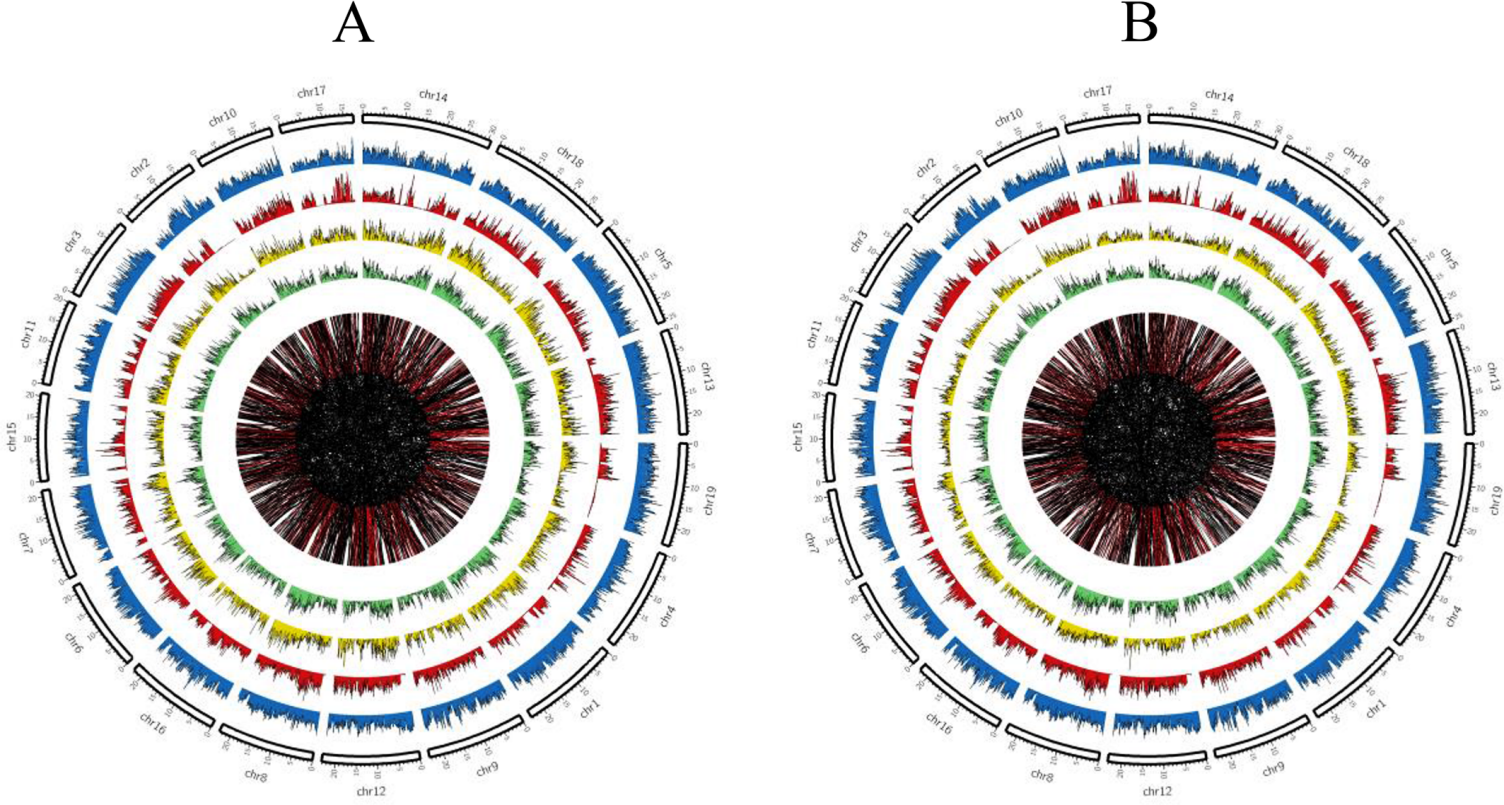
Different variation occurred in each chromosome.

We performed whole genome re-sequencing to evaluate the original reads (paired-end) and compared the reads with the reference genome. High reliability SNPs (3,692,777 in SBC and 3,823,464 SBBM) were identified with SAMtools software (Table 2). The transition percentage of SBC and SBBM was 69.66% and 69.53%, respectively. We also identified homozygous and heterozygous SNPs. The results showed that the homozygous and heterozygous ratios were above 27% and 71%, respectively (Fig 5). The distribution of SNPs, type, and zygosity of variants per chromosome in the two lines is shown in Table 3. The highest SNP number of 214,459 in SBC and 222,506 in SBBM was located on chr18 and the minimum number of 10,156 SNPs in SBC and 104,890 in SBBM on chr17. The percentage conversion to the same base type ranged from 68.6% to 71.6% and the percentage of conversion to a different type of base was between 28.4% and 31.75%.

**Table 2:** Total number of variants, type and zygosity of variants in each genotype.

Genotype^a^	SNP number^b^	Transition percentage^c^	Transversionpercentage^d^	Heterozygosity percentage^e^	Homozygositypercentage^f^
SBC	3,692,777	69.66%	30.34%	71.69%	28.31%
SBBM	3,823,464	69.53%	30.47%	72.74%	27.26%

^a^ Sample name.

^b^ The total number of SNPs.

^C^ Percentage conversion into same basetype.

^d^ Percentage conversion into different types of bases.

^e^ Heterozygous (two or more bases exist) percentage.

^f^ Homozygous (only one base exists) percentage.

**Table 3:** Distribution of SNPs, type and zygosity of variants per chromosome in the two grape strains.

Chr^a^	SNPs^b^		Trs^c^		Trv^d^		Het^e^		Hom^f^	
Genotype	SBC	SBBM	SBC	SBBM	SBC	SBBM	SBC	SBBM	SBC	SBBM
Chr1	198773	207314	68.6%	68.64%	31.35%	31.36%	76.71%	77.84%	23.29%	22.16%
Chr2	125954	130699	69.5%	69.57%	30.42%	30.43%	77.2%	77.88%	22.8%	22.12%
Chr3	169113	174349	70.4%	70.19%	29.53%	29.81%	68.33%	69.43%	31.69%	30.57%
Chr4	165317	172309	69.9%	69.79%	30.04%	30.21%	74.92%	75.97%	25.08%	24.03%
Chr5	225200	231442	69.7%	69.69%	30.21%	30.31%	74.65%	75.59%	26.35%	24.41%
Chr6	184826	193126	68.9%	68.87%	31.04%	31.13%	71.56%	72.48%	28.44%	27.52%
Chr7	161183	168199	69.1%	69.05%	30.82%	30.95%	72.03%	73.12%	27.97%	26.88%
Chr8	196361	202887	68.7%	68.73%	31.25%	31.27%	64.95%	65.71%	35.05%	34.29%
Chr9	196258	203205	69.8%	69.76%	30.16%	30.24%	70.58%	71.86%	29.42%	28.14%
Chr10	149493	154405	69.8%	69.75%	30.11%	30.25%	66.6%	67.68%	33.4%	32.14%
Chr11	146092	152081	70.4%	70.14%	29.58%	29.86%	75.55%	76.4%	24.45%	23.6%
Chr12	185733	190987	69.4%	69.35%	30.57%	30.65%	65.96%	67.16%	34.04%	32.84%
Chr13	214826	222976	69.2%	69.09%	30.73%	30.91%	71.5%	73.03%	28.5%	26.97%
Chr14	201949	208926	69.5%	69.49%	30.45%	30.51%	73.3%	74.25%	26.7%	25.74%
Chr15	140970	146118	70.8%	70.69%	29.16%	29.31%	78.53%	79.51%	21.47%	20.49%
Chr16	186206	192863	70.0%	69.98%	29.91%	30.02%	71.43%	72.56%	28.57%	27.44%
Chr17	101565	104890	71.6%	71.25%	28.4%	28.75%	64.37%	64.98%	35.63%	35.02%
Chr18	214459	222506	68.9%	68.79%	31.07%	31.21%	63.88%	64.58%	36.12%	35.42%
Chr19	182516	191064	69.2%	69.17%	30.75%	30.83%	85.96%	86.92%	14.06%	13.08%
**Total**	3346794	3347036	69.6%	69.58%	30.29%	30.42%	72.00%	73.00%	28.00%	27.00%

^a^Chromosome.

^b^The total number of SNPs.

^c^Percentage conversion into the same basetype.

^d^Percentage conversion into different types of bases.

^e^Heterozygous (two or more bases exist) percentage.

^f^Homozygous (only one base exists) percentage.

The changes to the sequences occurred in the intergenic, exon, and intronic regions. Table 4 shows that 61.02% and 62.13% of SNPs were in the intergenic region, 5.65% and 5.46% SNPs were in the exon region, and 33.33% and 32.41% SNPs were in the intron region in SBC and SBBM, respectively. The number of SNPs was 100times higher than the number of SVs in exons and the number of SNPs in the intergenic and intronic regions was50-fold and 30-fold higher in SBC and SBBM, respectively.

**Table 4:** Different component statistics of SNP, SV in the genomes.

Sample	Type	Intergenic	Exon	Intron
SBC	fSNP	2,253,327	208497	1,231,188
	SV	36,239	2,120	42,864
SBBM	SNP	2,375,628	208,692	1,239,392
	SV	40,791	2,157	42,853

Numbers of SNPs and SVslocated in exon, intron and intergenic regions.

**Fig 6:**
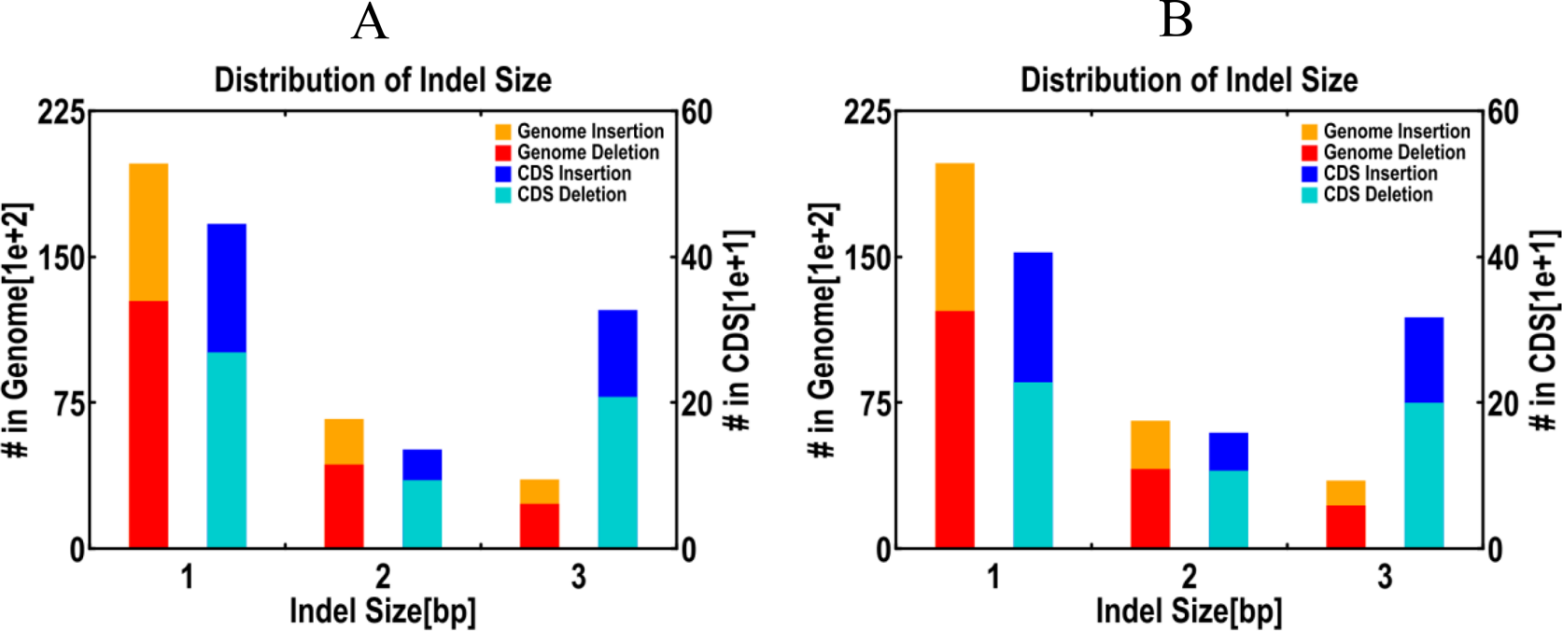
Diferent InDel sizes distribution and frameshift mutation between the whole genome and CDS region.

We considered insertions, deletions, inversions, intra-chromosome transfers and inter-chromosome transfers as SVs. The distribution of SVs and the type of variants per chromosome in the two genotypes (Table 5) shows that the proportion of insertions and deletions was greater than 95%, whereas other SVs represented less than 5%. The indel size distribution and frame shift mutation in the whole genome and coding region (CDS) is shown in Fig 6. The number of single-bp (base pair) indels, two-bp indels, three-bp indels, insertions and deletions that occurred in the SBC and SBBM whole genomes was extremely similar, and were about 7,500 and 12,500, 2,500 and 4,500, and 1,500 and 2,500, respectively and those in the CDS region were about 750 and 850, 240 and 110, and 200 and 150, respectively.

**Table 5:** Distribution of SV and the type of variants per chromosome in the two grape lines.

Chr^a^	SV Number^b^		INS^c^(%)		DEL^d^(%)		IDEL^e^(%)		INV^f^(%)		ITX^g^(%)		CTX^h^(%)	
genotype	SBC	SBBM	SBC	SBBM	SBC	SBBM	SBC	SBBM	SBC	SBBM	SBC	SBBM	SBC	SBBM
Chr1	5,043	5,211	60.72	58.45	35.95	38.98	0.48	0.35	0.18	0.13	0.38	0.44	2.3	1.65
Chr2	2,831	3,017	61.14	58.67	35.96	38.78	0.53	0.63	0.04	0.03	0.57	0.7	1.77	1.19
Chr3	3,087	3,276	58.79	56.26	38.1	40.29	0.71	0.98	0.1	0.03	0.75	0.85	1.55	1.59
Chr4	4,168	4,425	58.71	56.14	39.04	41.79	0.65	0.54	0.07	0.11	0.55	0.45	0.98	0.97
Chr5	5,239	5,363	61.73	59.46	36.17	38.56	0.59	0.67	0.1	0.13	0.61	0.54	0.8	0.63
Chr6	4,793	5,194	61.53	58.59	36.72	39.89	0.42	0.52	0.06	0.12	0.38	0.25	0.9	0.64
Chr7	4,006	4,254	59.94	57.36	37.94	40.69	0.6	0.63	0.1	0.12	0.65	0.63	0.77	0.56
Chr8	5,408	5,795	61.54	57.01	37.15	41.57	0.55	0.57	0.15	0.16	0.37	0.4	0.24	0.29
Chr9	3,679	3,910	62.27	59.23	35.61	38.39	0.63	0.61	0.22	0.26	0.95	1.07	0.33	0.43
Chr10	3,345	3,490	59.52	55.59	35.61	40.57	0.69	0.43	0.36	0.29	0.75	0.49	3.08	2.64
Chr11	3,646	3,762	58.72	55.69	36.94	40.16	0.8	0.53	0.14	0.16	0.58	0.43	2.83	3.03
Chr12	4,320	4,509	57.52	55.98	37.52	39.59	0.79	0.6	0.02	0.04	1.02	0.93	3.12	2.86
Chr13	4,739	5,043	57.65	54.71	38.34	40.97	0.61	0.83	0.08	0.06	0.8	0.93	2.51	2.5
Chr14	5,053	5,277	58.24	56.07	37.92	40.38	0.85	0.76	0.12	0.15	0.85	0.72	2.02	1.91
Chr15	2,913	2,980	58.15	56.54	36.63	39.09	0.79	0.74	0.27	0.2	0.65	0.67	3.5	2.75
Chr16	3,475	3,751	61.29	56.81	34.45	39.3	0.78	0.69	0.06	0.13	0.86	0.88	2.56	2.19
Chr17	2,608	2,824	56.29	54.07	40.87	42.53	0.5	0.64	0.12	0.11	0.46	0.42	1.76	2.23
Chr18	5,739	6,464	58.18	54.81	38.84	42.39	0.54	0.51	0.07	0.12	0.84	0.79	1.53	1.38
Chr19	3,409	3,614	61.87	58.83	34.85	38.21	0.79	0.8	0.15	0.11	0.88	0.75	1.47	1.3
Total	77,501	82,159	59.67	56.86	37.08	40.11	0.65	0.63	0.13	0.13	0.68	0.65	1.79	1.62

^a^Chromosome genotype.

^b^Number of SVs.

^c^Percentage of insertion type.

^d^Percentage of deletion type.

^e^Percentage of the deletion that contained an insertion.

^f^Percentage of the inversion type.

^g^Percentage of intra-chromosome transfer.

^h^Percentage of inter-chromosome transfer.

The gene: SNP (non-synonymous) ratio in SBC and SBBM (Table 6) varied from21,251 to 118,778 and from 21,235 to 118,643, respectively, whereas the gene: SNP (≥10 non-synonymous) ratio was between 3,332 to 53,295 and 3,298 to 53,051, respectively. The gene: indel (with frame-shift) ratio was 630 to 661 and 626 to 650, respectively, whereas the gene: SV (with SVs) ratio was 1,922 to 2,120 and 1,931 to 2,157, respectively.

**Table 6:** Genotype-specific statistical differences between the various categories of gene mutation.

Sample	Genes:SNPs^a^(with non-synonymous)	Genes:SNPs^b^(with(> = 10) non-synonymous)	Genes:InDels^c^(with frame-shift)	Genes:SVs^d^(withSVs)
SBC	21,251:118,778	3,332:53,295	630:661	1,922:2,120
SBBM	21,235:118,643	3,298:53,051	626:650	1,931:2,157

^a^Number of genes with non-synonymous mutations: Number of the corresponding SNPs.

^b^Number of SNPs with non-synonymous mutations where the number of SNPs is greater than 10: the number of the corresponding SNPs.

^c^The number of genes containing frame shift mutations (InDels): the number of the corresponding InDels.

^d^The number of genes containing variants of SVs: the corresponding number of SVs.

We consider between 20,000 and 25,000 bp per SNP to be ahigh-density SNP region. Compared with the reference genome, many high-density SNP regions were found on different chromosomes (Table 7). Chr17 (six regions) and chr2 (four regions) had more high-density SNP regions than the other chromosomes. Twelve high-density SNP regions and 15 low-density SNP regions were detected in SBC, whereas 10 high-density SNP regions and 14 low-density SNP regions were found in SBBM. In addition, SBC had two high-density SNP regions and one low-density SNP region that SBBM did not possess.

**Table 7:** Recognizable high- or low-density SNP regions compared between SBC and SBBM.

SBC					SBBM				
Chromosome	Region^a^		SNPnumber^b^	Highorlow^c^	Chromosome	Region^a^		SNPnumber^b^	High or low^c^
chr2	7,280,001	7,660,000	16	H	chr2	7,760,001	8,560,000	28	H
chr2	9,120,001	10,500,000	59	H	chr2	9,120,001	10,080,000	48	H
chr2	11,120,001	11,880,000	36	L	chr2	11,120,001	11,880,000	38	L
chr2	14,200,001	14,540,000	17	L	chr2	14,200,001	14,540,000	17	L
chr3	2,820,001	3,180,000	17	L	chr3	2,840,001	3,180,000	16	L
chr4	10,120,001	10,600,000	20	H	chr4	10,160,001	10,620,000	19	H
chr7	1,500,001	3,300,000	89	L	chr7	1,500,001	3,300,000	88	L
chr7	18,460,001	18,880,000	19	H	chr7	18,440,001	18,880,000	19	H
chr9	14,560,001	14,960,000	20	L	chr9	14,540,001	14,960,000	21	L
chr14	11,800,001	12,660,000	43	L	Chr14	11,800,001	12,660,000	43	L
chr14	19,020,001	19,660,000	29	L	Chr14	19,020,001	19,640,000	27	L
chr14	27,000,001	27,400,000	18	H	chr14	27,000,001	27,400,000	19	H
chr15	6,140,001	7,100,000	34	H	Chr15	6,140,001	7,100,000	39	H
chr15	7,620,001	7,960,000	17	L	Chr15	7,620,001	7,960,000	17	L
chr16	10,100,001	10,540,000	22	H	Chr16	10,180,001	10,540,000	18	H
chr16	15,060,001	15,400,000	17	L	Chr16	15,060,001	15,400,000	17	L
chr16	19,660,001	20,500,000	39	L	Chr16	19,660,001	20,500,000	34	L
chr17	200,001	680,000	24	L	Chr17	200,001	680,000	24	L
chr17	4,900,001	5,320,000	21	L	Chr17	4,920,001	5,320,000	20	L
chr17	7,120,001	7,520,000	20	L	Chr17	7,100,001	7,520,000	21	L
chr17	11,800,001	12,760,000	34	H	Chr17	12,020,001	12,760,000	27	H
chr17	13,040,001	13,520,000	24	H	Chr17	13,080,001	13,520,000	22	H
chr17	14,780,001	15,340,000	27	H	Chr17	14,780,001	15,340,000	27	H
chr19	14,060,001	14,520,000	22	L	Chr19	14,080,001	14,560,000	24	L
chr5	13,340,001	13,660,000	16	H					
chr11	7,060,001	7,400,000	17	L					
chr18	15,780,001	16,100,000	16	H					

^a^Region on chromosome.

^b^SNP number on chromosome region.

^c^High or low SNP compared with the reference genome.

### Gene categories, functional annotation and differences in genes

**Fig 7:**
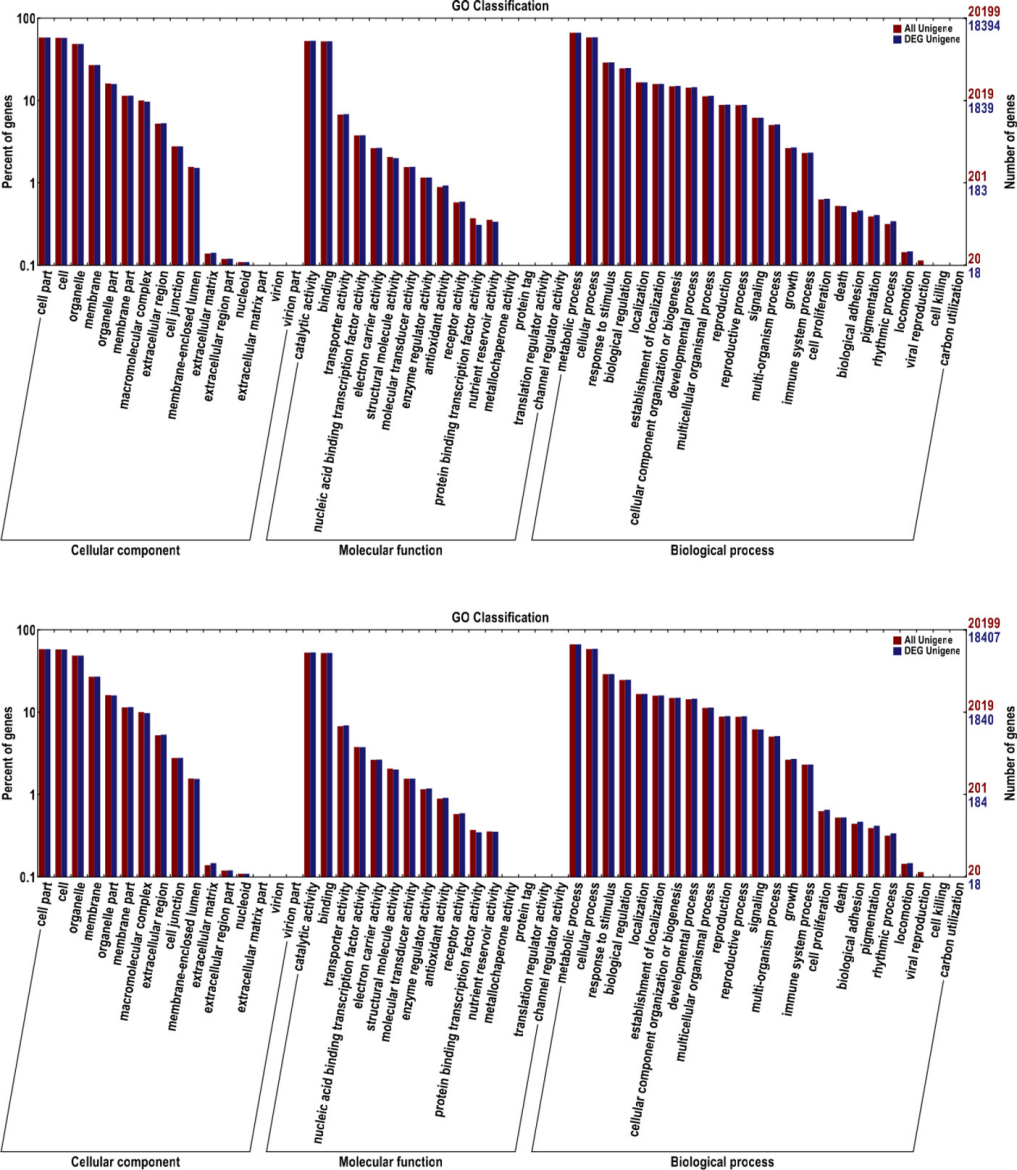
GO classify for differential UniGenes between ‘SBC’ (up) and ‘SBBM’(down).

Non-synonymous (NS) SNP mutations and frameshifts (F) caused by missing bases not in a multiple of three bases, as well as SVs caused by indels, transversions, and transitions might influence the expression of the relevant protein. All tested and functional annotated genes were classified into gene ontology (GO) categories (Fig 7), which mainly divided the genes into three categories: cellular components, molecular function, and biological process. The GO enrichment classification suggested that the genes from the cellular component, molecular function and biological process categories could be divided into 18, 18 and 26 groups, respectively.

**Fig 8:**
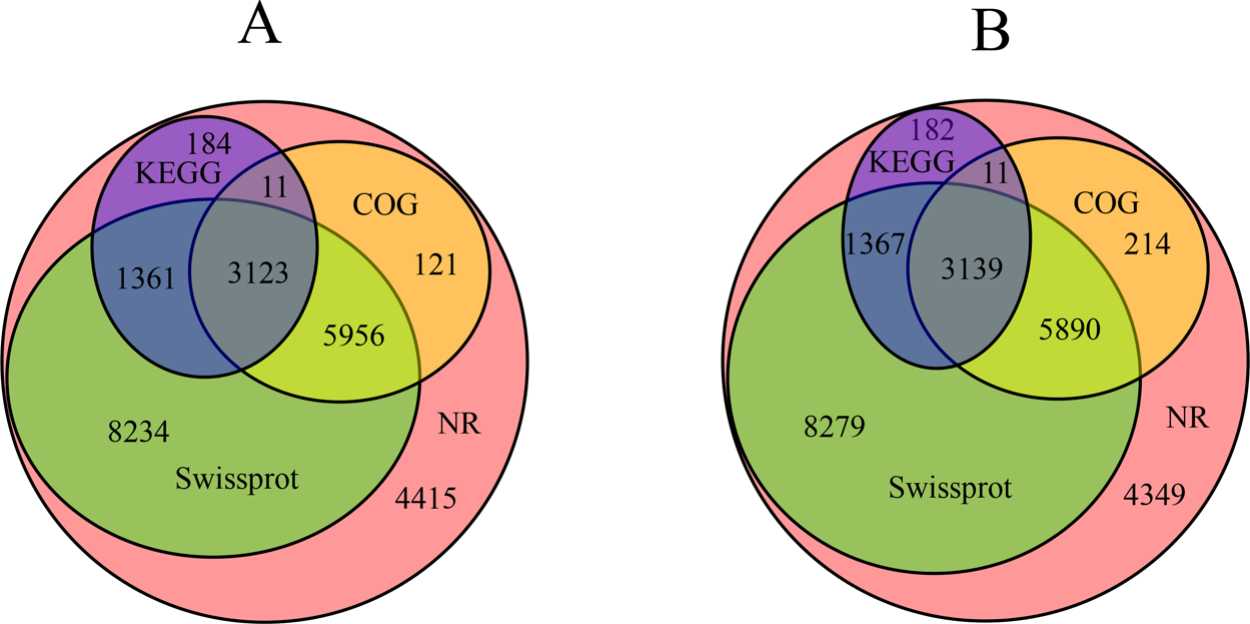
Homology analyze of ‘SBC’ (A) and ‘SBBM’ (B) relative to reference genome.

The genes annotated by BLASTx, with a threshold of 10^-5^, were recorded in four public databases including the NCBI non-redundant database (NR), the Swissprot protein database, the Clusters of Orthologous Groups of proteins database (COG) and the Kyoto Encyclopedia of Genes and Genomes (KEGG) (S1 Table). A total of 23,405 genes in SBC and 23,431 genes in SBBM were functionally annotated (Fig 8). All the genes were recorded in the NR database, and the genes annotated in the four databases were quantitatively similar. 5,956 genes in SBC and 5,890 genes in SBBM were annotated by all four databases.

**Fig 9:**
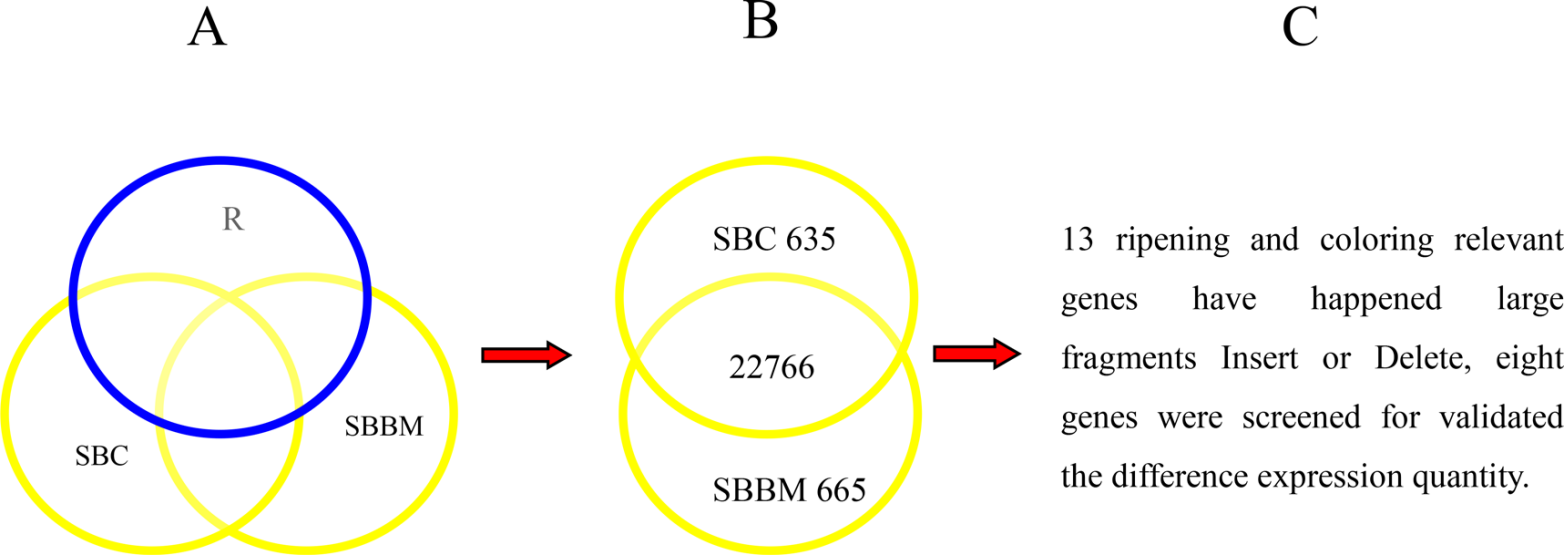
Flowchart for finding the causative different genes for ripening and coloring.

Compared with the reference genome, different homologous unigenes associated with ripening and colour was screened in the two cultivated lines. Thirteen proteins associated with ripening and colour were detected as S/NS, SV or F, these genes encoded MYB, bHLH or WD40 proteins or were involved in ETH, anthocyanidin, ABA, CTK, auxin, GA, BR, sugar, polyamine, and salicylic acid pathways. Ninety unigenes in SBC and 76 unigenes in SBBM were detected as S/NS, SV or F (S3 Table). Among these, Myb-related protein 3R-1 (GSVIVT01011986001) and Myb-related protein 306 (GSVIVT01008005001) occurred as S/NS only in SBBM. In total, 635 SBC-specific genes and 635 SBBM-specific genes were screened. Thirteen genes related to ripening, i.e., *CHSY*, *MYBF*, *3MAT*, *ERF5*, *ERF03*, *AI5L2*, *SEP1*, *STC*, *ERDL5*, *PT106*, *AX10A*, *AXX15*, and *NCED6* (Table 8) occurred with a large Indel fragment. Among these, differences in expression for eight genes were validated by qRT-PCR (Fig 9).

**Table 8:** Thirteen large-fragment InDels in candidate genes related to ripening.

Annotated protein^a^	Gene & protein ID^b^	Gene ID &Chromosome^c^	Change-SBC^d^	Change-SBBM^e^
**—**	**—**	**—**	**NS**	**NS/Sv**
Chalcone synthase	sp|P51090|CHSY_VITVI	GSVIVT01032968001 in chr14	24,673,846 T AA	24,673,846 T AA
halcone synthase	sp|P51090|CHSY_VITVI	GSVIVT01032968001 in chr14	24,673,881 A CC	24,673,881A CC
Chalcone synthase	sp|P51090|CHSY_VITVI	GSVIVT01032968001 in chr14	24,673,978 C TT	24,673,978 C TT
Chalcone synthase	sp|P51090|CHSY_VITVI	GSVIVT01032968001 in chr14	24,674,524 T CC	24,674,194 T CY
Chalcone synthase	sp|P51090|CHSY_VITVI	GSVIVT01032968001 in chr14	—	24,674,524 T CC
Chalcone synthase	sp|P51090|CHSY_VITVI	GSVIVT01032968001 in chr14	—	24,675,008 C TT
Chalcone synthase	sp|P51090|CHSY_VITVI	GSVIVT01032968001 in chr14	—	DEL: 13,541 from 24,674,193 to 24,687,822
—	—	—	**NS/Sv/F**	**NS**
Myb-family transcription factor	sp|Q700D9|MYF _ARATH	GSVIVT01032776001 in chr13	838,415 C GG	838,867 C GG
Myb-family transcription factor	sp|Q700D9|MYF _ARATH	GSVIVT01032776001 in chr13	838,558 C AM	838,900 T AW
Myb-family transcription factor	sp|Q700D9|MYF _ARATH	GSVIVT01032776001 in chr13	838,867 C GG	839,256 T CC
Myb-family transcription factor	sp|Q700D9|MYF _ARATH	GSVIVT01032776001 in chr13	838,900 T AW	839,647 T CY
Myb-family transcription factor	sp|Q700D9|MYF _ARATH	GSVIVT01032776001 in chr13	839,256 T CC	844,828 T CC
Myb-family transcription factor	sp|Q700D9|MYF _ARATH	GSVIVT01032776001 in chr13	839,647 T CY	844,891T CY
Myb-family transcription factor	sp|Q700D9|MYF _ARATH	GSVIVT01032776001 in chr13	839,929 G AR	—
Myb-family transcription factor	sp|Q700D9|MYF _ARATH	GSVIVT01032776001 in chr13	840,005 C TY	—
Myb-family transcription factor	sp|Q700D9|MYF _ARATH	GSVIVT01032776001 in chr13	840,080 C AM	—
Myb-family transcription factor	sp|Q700D9|MYF _ARATH	GSVIVT01032776001 in chr13	844,891T CY	—
Myb-family transcription factor	sp|Q700D9|MYF _ARATH	GSVIVT01032776001 in chr13	DEL4: from 838,413 to 838,420	—
Myb-family transcription factor	sp|Q700D9|MYF _ARATH	GSVIVT01032776001 in chr13	INS 2: from 84,0284to84,0286	—
Myb-family transcription factor	sp|Q700D9|MYF _ARATH	GSVIVT01032776001 in chr13	INS 1: from 84,4790to 84,4799	—
—	—	—	**NS**	**NS/Sv**
Malonyl-coenzyme A	sp|Q8GSN8|3MAT_DAHPI	GSVIVT01000461001 in chr12	8,202,818 A TT	8,202,818 A TT
Malonyl-coenzyme A	sp|Q8GSN8|3MAT_DAHPI	GSVIVT01000461001 in chr12	8,202,844 T CY	8,203,022 G AA
Malonyl-coenzyme A	sp|Q8GSN8|3MAT_DAHPI	GSVIVT01000461001 in chr12	8,203,022 G AA	8,203,027 A CM
Malonyl-coenzyme A	sp|Q8GSN8|3MAT_DAHPI	GSVIVT01000461001 in chr12	8,203,027 A CM	8,203,486 C TT
Malonyl-coenzyme A	sp|Q8GSN8|3MAT_DAHPI	GSVIVT01000461001 in chr12	8,203,207 A TW	8,210,389 G CS
Malonyl-coenzyme A	sp|Q8GSN8|3MAT_DAHPI	GSVIVT01000461001 in chr12	8,203,216 A TW	8,210,633 A GR
Malonyl-coenzyme A	sp|Q8GSN8|3MAT_DAHPI	GSVIVT01000461001 in chr12	8,203,346 C TY	—
Malonyl-coenzyme A	sp|Q8GSN8|3MAT_DAHPI	GSVIVT01000461001 in chr12	8,203,486 C TT	—
Malonyl-coenzyme A	sp|Q8GSN8|3MAT_DAHPI	GSVIVT01000461001 in chr12	8,210,571 T CY	—
Malonyl-coenzyme A	sp|Q8GSN8|3MAT_DAHPI	GSVIVT01000461001 in chr12	8,210,633 A GR	—
Malonyl-coenzyme A	sp|Q8GSN8|3MAT_DAHPI	GSVIVT01000461001 in chr12	—	DEL25,483: from 829,838 to 8,235,429
—	—	—	**NS**	**NS/Sv**
Ethylene-responsive transcription factor	sp|Q40478|ERF5_TOBAC	GSVIVT01013934001 in chr16	7,012,358 G AR	7,011,626 T CC
Ethylene-responsive transcription factor	sp|Q40478|ERF5_TOBAC	GSVIVT01013934001 in chr16	7,012,365 A GR	7,012,358 G AR
Ethylene-responsive transcription factor	sp|Q40478|ERF5_TOBAC	GSVIVT01013934001 in chr16	7,012,405 T GK	7,025,216 G CS
Ethylene-responsive transcription factor	sp|Q40478|ERF5_TOBAC	GSVIVT01013934001 in chr16	7,020,752 G CS	7,025,318 C TT
Ethylene-responsive transcription factor	sp|Q40478|ERF5_TOBAC	GSVIVT01013934001 in chr16	7,025,275 G AA	7,025,356 T CY
Ethylene-responsive transcription factor	sp|Q40478|ERF5_TOBAC	GSVIVT01013934001 in chr16	7,025,356 T CY	7,025,450 C TY
Ethylene-responsive transcription factor	sp|Q40478|ERF5_TOBAC	GSVIVT01013934001 in chr16	7,025,751 C TY	7,025,692 A GR
Ethylene-responsive transcription factor	sp|Q40478|ERF5_TOBAC	GSVIVT01013934001 in chr16	7,025,772 A CC	—
Ethylene-responsive transcription factor	sp|Q40478|ERF5_TOBAC	GSVIVT01013934001 in chr16	7,025,782 G CC	—
Ethylene-responsive transcription factor	sp|Q40478|ERF5_TOBAC	GSVIVT01013934001 in chr16	—	DEL8,818: from 7,011,486 to 7,020,308
—	—	—	**—**	**Sv**
Ethylene-responsive transcription factor	sp|Q94AW5|ERF03_ARATH	GSVIVT01026334001 in chr4	—	DEL12,460: from 14,176,993 to 14,189,460
—	—	**—**	**Sv**	
ABSCISIC ACID-INSENSITIVE 5-like protein	sp|Q9LES3|AI5L2_ARATH	GSVIVT01033216001 in chr4	DEL7,576:from 9,840,430 to 9,847,952	—
—	—	—	S	S/Sv/F
Developmental protein SEPALLATA 1	sp|P29382|SEP1_ARATH	GSVIVT01008139001 in chr17	—	DEL4: from 5,464,406 to 5,464,422
—	—	—	**NS**	**NS/Sv**
Sugar carrier protein C	sp|Q41144|STC_RICCO	GSVIVT01036440001 in chr14	21,932,262 A GG	21,932,208 C TY
Sugar carrier protein C	sp|Q41144|STC_RICCO	GSVIVT01036440001 in chr14	21,932,385 T CY	21,932,226 G AR
Sugar carrier protein C	sp|Q41144|STC_RICCO	GSVIVT01036440001 in chr14	21,932,496 C TY	21,932,253 A GR
Sugar carrier protein C	sp|Q41144|STC_RICCO	GSVIVT01036440001 in chr14	21,939,640 A GR	21,932,262 A GG
Sugar carrier protein C	sp|Q41144|STC_RICCO	GSVIVT01036440001 in chr14	21,939,792 T GK	21,932,389 A CM
Sugar carrier protein C	sp|Q41144|STC_RICCO	GSVIVT01036440001 in chr14	21,940,202 T GK	21,932,496 C TY
Sugar carrier protein C	sp|Q41144|STC_RICCO	GSVIVT01036440001 in chr14	21,940,212 C AM	21,933,149 C AA
Sugar carrier protein C	sp|Q41144|STC_RICCO	GSVIVT01036440001 in chr14	21,940,573 T CY	21,939,727 G AR
Sugar carrier protein C	sp|Q41144|STC_RICCO	GSVIVT01036440001 in chr14	21,940,659 A CC	21,939,792 T GK
Sugar carrier protein C	sp|Q41144|STC_RICCO	GSVIVT01036440001 in chr14	21,940,702 G CS	21,940,059 A CM
Sugar carrier protein C	sp|Q41144|STC_RICCO	GSVIVT01036440001 in chr14	21,941,645 C TY	21,940,118 G TK
Sugar carrier protein C	sp|Q41144|STC_RICCO	GSVIVT01036440001 in chr14	21,941,697 C TY	21,940,202 T GK
Sugar carrier protein C	sp|Q41144|STC_RICCO	GSVIVT01036440001 in chr14	21,941,924 A GR	21,940,212 C AM
Sugar carrier protein C	sp|Q41144|STC_RICCO	GSVIVT01036440001 in chr14	21,941,948 A GR	21,940,573 T CY
Sugar carrier protein C	sp|Q41144|STC_RICCO	GSVIVT01036440001 in chr14	21,942,036 C AM	21,940,659 A CC
Sugar carrier protein C	sp|Q41144|STC_RICCO	GSVIVT01036440001 in chr14	21,942,092 A TW	21,940,702 G CS
Sugar carrier protein C	sp|Q41144|STC_RICCO	GSVIVT01036440001 in chr14	—	DEL3,328: from 21,935,542 to 21,938,876
—	—	—	**NS/Sv**	**NS**
Sugar transporter	sp|Q3ECP7|ERDL5_ARATH	GSVIVT01022025001 in chr14	4,161,687 A GR	4,161,760 G AR
Sugar transporter	sp|Q3ECP7|ERDL5_ARATH	GSVIVT01022025001 in chr14	4,161,760 G AR	4,161,928 G CS
Sugar transporter	sp|Q3ECP7|ERDL5_ARATH	GSVIVT01022025001 in chr14	4,161,906 T CY	4,161,979 C TT
Sugar transporter	sp|Q3ECP7|ERDL5_ARATH	GSVIVT01022025001 in chr14	4,161,928 G CS	4,162,107 C TT
Sugar transporter	sp|Q3ECP7|ERDL5_ARATH	GSVIVT01022025001 in chr14	4,161,979 C TT	4,164,395 C TT
Sugar transporter	sp|Q3ECP7|ERDL5_ARATH	GSVIVT01022025001 in chr14	4,162,107 C TT	4,164,776 T GG
Sugar transporter	sp|Q3ECP7|ERDL5_ARATH	GSVIVT01022025001 in chr14	—	4,164,823 G AR
Sugar transporter	sp|Q3ECP7|ERDL5_ARATH	GSVIVT01022025001 in chr14	—	4,164,829 G CC
Sugar transporter	sp|Q3ECP7|ERDL5_ARATH	GSVIVT01022025001 in chr14	DEL1,903: from 4,164,061 to 4,165,992	—
—	—	—	**NS**	**NS/Sv**
sugar phosphate/phosphate translocator	sp|Q8H184|PT106_ARATH	GSVIVT01020780001 in chr12	2,066,209 A CM	2,066,209 A CM
sugar phosphate/phosphate translocator	sp|Q8H184|PT106_ARATH	GSVIVT01020780001 in chr12	2,068,143 A TT	2,066,236 C TY
sugar phosphate/phosphate translocator	sp|Q8H184|PT106_ARATH	GSVIVT01020780001 in chr12	2,068,152 C TY	2,068,143 A TT
sugar phosphate/phosphate translocator	sp|Q8H184|PT106_ARATH	GSVIVT01020780001 in chr12	2,071,379 C TY	2,068,152 C TY
sugar phosphate/phosphate translocator	sp|Q8H184|PT106_ARATH	GSVIVT01020780001 in chr12	2,071,382T CY	2,071,379 C TY
sugar phosphate/phosphate translocator	sp|Q8H184|PT106_ARATH	GSVIVT01020780001 in chr12	—	2,071,382 T CY
sugar phosphate/phosphate translocator	sp|Q8H184|PT106_ARATH	GSVIVT01020780001 in chr12	—	DEL17,541: from 2,095,192 to 2,112,841
—	—	—	**NS**	**NS/Sv**
Auxin-induced protein	sp|P33080|AX10A_SOYBN	GSVIVT01023911001 in chr3	2,525,484 A TW	2,525,618 C AM
Auxin-induced protein	sp|P33080|AX10A_SOYBN	GSVIVT01023911001 in chr3	2,525,502 T AW	2,525,636 G TT
Auxin-induced protein	sp|P33080|AX10A_SOYBN	GSVIVT01023911001 in chr3	2,525,618 C AM	2,525,670 T CC
Auxin-induced protein	sp|P33080|AX10A_SOYBN	GSVIVT01023911001 in chr3	2,525,636 G TT	2,525,815 C GS
Auxin-induced protein	sp|P33080|AX10A_SOYBN	GSVIVT01023911001 in chr3	2,525,670 T CC	2,525,831 T AW
Auxin-induced protein	sp|P33080|AX10A_SOYBN	GSVIVT01023911001 in chr3	2,525,815 C GS	2,525,874 T CY
Auxin-induced protein	sp|P33080|AX10A_SOYBN	GSVIVT01023911001 in chr3	2,525,831 T AW	—
Auxin-induced protein	sp|P33080|AX10A_SOYBN	GSVIVT01023911001 in chr3	2,525,874 T CY	—
Auxin-induced protein	sp|P33080|AX10A_SOYBN	GSVIVT01023911001 in chr3	—	DEL7,836: from 2,525,478 to 2,533,405
—	—	—	**NS**	**NS/Sv**
.Auxin-induced protein	sp|P33082|AXX15_SOYBN	GSVIVT01024129001 in chr3	888,891 C TY	888,891 C TY
Auxin-induced protein	p|P33082|AXX15_SOYBN	GSVIVT01024129001 in chr3	888,934 G TK	—
Auxin-induced protein	p|P33082|AXX15_SOYBN	GSVIVT01024129001 in chr3	—	DEL9,447: from 886,035 to 895,516
—	—	—	**NS**	**NS/Sv**
9-cis-epoxycarotenoid dioxygenase	sp|Q9LRM7|NCED6_ARATH	GSVIVT01029057001 in chr5	11,589,359 G CC	11,589,359 G CC
9-cis-epoxycarotenoid dioxygenase	sp|Q9LRM7|NCED6_ARATH	GSVIVT01029057001 in chr5	11,589,455 T GK	11,589,444 T GK
9-cis-epoxycarotenoid dioxygenase	sp|Q9LRM7|NCED6_ARATH	GSVIVT01029057001 in chr5	11,589,539 T GG	11,589,455 T GK
9-cis-epoxycarotenoid dioxygenase	sp|Q9LRM7|NCED6_ARATH	GSVIVT01029057001 in chr5	11,589,680 C TT	11,589,680 C TX
9-cis-epoxycarotenoid dioxygenase	sp|Q9LRM7|NCED6_ARATH	GSVIVT01029057001 in chr5	11,589,818 T GG	11,589,818 T GG
9-cis-epoxycarotenoid dioxygenase	sp|Q9LRM7|NCED6_ARATH	GSVIVT01029057001 in chr5	11,589,955 T AA	11,589,955 T AA
9-cis-epoxycarotenoid dioxygenase	sp|Q9LRM7|NCED6_ARATH	GSVIVT01029057001 in chr5	11,589,956 T CY	11,589,956 T CY
9-cis-epoxycarotenoid dioxygenase	sp|Q9LRM7|NCED6_ARATH	GSVIVT01029057001 in chr5	11,590,009 G TK	11,590,009 G TK
9-cis-epoxycarotenoid dioxygenase	sp|Q9LRM7|NCED6_ARATH	GSVIVT01029057001 in chr5	11,590,035 C AM	11,590,283 G AR
9-cis-epoxycarotenoid dioxygenase	sp|Q9LRM7|NCED6_ARATH	GSVIVT01029057001 in chr5	11,590,697 C TY	11,590,829 C TT
9-cis-epoxycarotenoid dioxygenase	sp|Q9LRM7|NCED6_ARATH	GSVIVT01029057001 in chr5	11,590,698 G AR	—
9-cis-epoxycarotenoid dioxygenase	sp|Q9LRM7|NCED6_ARATH	GSVIVT01029057001 in chr5	11,590,829 C TT	—
9-cis-epoxycarotenoid dioxygenase	sp|Q9LRM7|NCED6_ARATH	GSVIVT01029057001 in chr5	—	DEL15: from 11,591,007 to11,591,031

^a^The functional annotation of genes.

^b^Genes and proteins ID.

^c^Gene ID and chromosome position.

^d^Change occurred inSBC.

^e^Change occurred inSBBM. S/NS/Sv/F represents synonymous/non-synonymous/structure variation/frameshift variation.

### Genes on the transcriptional level validated by qRT-PCR

**Fig 10:**
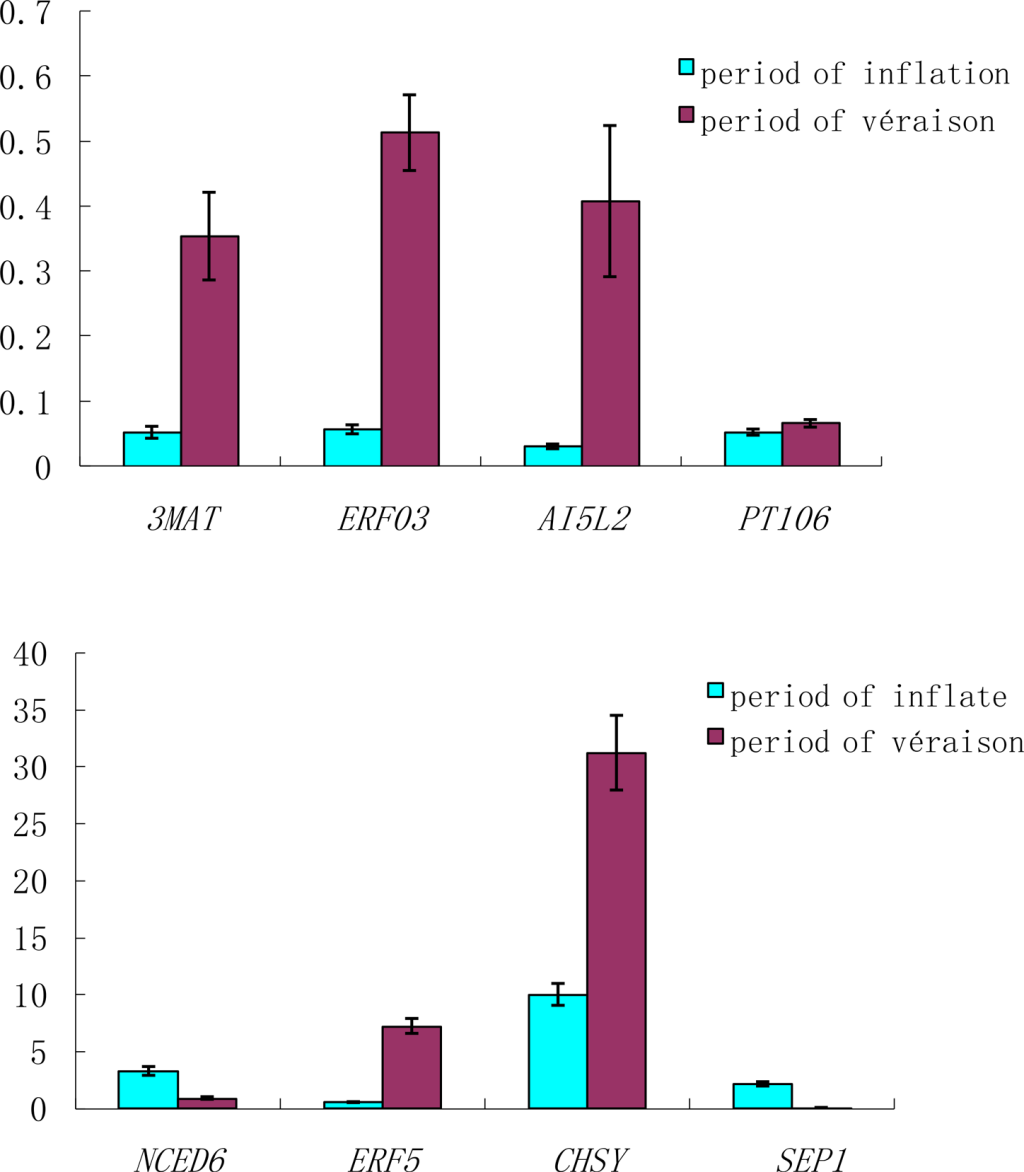
The expression of eight genes that potentially relate to SBBM grape ripening via qRT-PCR.

Eight genes that correlated with ripening and colour were detected and qRT-PCR was performed to validate their relative expression (Fig 10). The eight genes were *CHSY*, *3MAT*, *ERF5*, *ERF03*, *AI5L2*, *SEP1*, *PT106*, and *NCED6*.

The results showed that the expression of *CHSY*, *3MAT*, *ERF5*, *ERF03*, *AI5L2*, *SEP1*, and *PT106* was up-regulated and the expression of *SEP1* was down-regulated during véraison compared to during the green period (period of fruit inflation).

## Discussion

Sequence variation is commonly reflected by SNPs and SVs and small variations give rise to huge variations in horticultural traits in plants. Combined with a considerable number of SNPs and SVs in genes, proteins will be a major focus in future molecular biology-assisted breeding programs.

### SNP and SV analysis

The results here show that the ratios of SNPs identified in intergenic, exonic, or intronic regions were 61.0% and 62.1%, 5.6% and 5.5%, 33.4% and32.4%, in SBC and SBBM, respectively. Compared with *Arabidopsis*, rice [30, 31], and sorghum [32], the intergenic regions of grape genes contain more SNPs. Furthermore, compared with the reference genome, SBBM had more SNPs, transversions (Trvs) and heterozygous genes than SBC on each chromosome. Moreover, the several high- or low-density SNP regions distributed on the SBC and SBBM chromosomes will be useful for further mapping, particularly to identify markers that are closely linked to QTLs. Similarly, some regions, such as the region between 13.34 and 13.66 Mb on chromosome 5 (16 SNPs) and the region between 15.78 and 16.10 Mb on chr18 (16 SNPs), had a higher SNP density only in SBC. The region between 7.06 and 7.40 Mb on chr11 (17 SNPs) had a higher SNP density in SBBM than in SBC. In contrast with in other species, the number of SNPs per 0.1 Mb region was lower in our two grape culti-species, as beans contain about 650 SNPs per Mb in [33]. Moreover, some chromosomes did not have high-density SNP regions, i.e., chr1, chr6, chr8, chr9, chr10, chr12, and chr13 in SBC, and chr1, chr5, chr6, chr8, chr9, chr10, chr11, chr12, chr13, and chr18 in SBBM.

Research into grape berry colour has identified that the berry colour locus is a cluster of Myb and Myb-like genes within a 200-kb region located on chr2 [34]. Compared with the reference genome, one high-density SNP region was present on chr2. These results might be related to early colouring and early ripening in SBBM. The distribution of indel sizes shows that there were more insertions than deletions in the two genomes compared with the reference genome. Moreover, single-base indels accounted for a larger proportion than two- and three-base indels in the two cultivars. Interestingly, three-base indels in the CDS were present at a higher frequency than two-base indels.

The GO enrichment analysis provided 62 categories in cellular components, molecular functions and biological processes that were significantly overrepresented. Notably, genes related to pigmentation, growth, and carbon-use, as well as the response to stimuli was found within the biological processes. Unigene homology analysis suggested that all the annotated unigenes were present in the NR database, followed by the Swissprot database, although the Swissprot database was more reliable, due to its manually entered homology unigenes. In total, 635 unigenes in SBC and 665 unigenes in SBBM were screened. Among these, we filtered 90 unigenes in SBC and 76 unigenes in SBBM that were related to colouring and ripening. Changes (NS, SV or Fin SBC and SBBM) were found in the unigenes for ABA (23 and 23), ETH (8 and 7), BRs (11 and 5), GAs (6 and 3), sugar (5 and 1), IAAs (14 and 12), CTKs (4 and 3), anthocyanidins (1 and 1), MYB (1 and 3), bHLH (1 and 1), WD40 (1 and 2), salicylic acid (13 and 12) and polyamine (2 and 4), respectively (S3 Table). These results might be mainly related to colour, early ripening, and disease resistance compared with the ‘Pinot noir’ reference genome, which shows the agronomic traits of lighter colouration, mid-maturation and weak disease resistance in Europe and America [35]. In addition to the functionally annotated genes, many new unnamed unigenes were detected that require further exploration to determine gene function.

### The *NCED6* gene appears to be involved in ABA biosynthesis and ABA might promote anthocyanin accumulation

The ABA concentration results show that the content of ABA increased at about the time of véraison [36]. Previous studies have shown that the content of ABA increases at the start of sugar accumulation and then reaches a peak two to three weeks later, after which the ABA content declines as the fruit approaches ripeness [37,38]. Zhang et al. found that the ABA content in grapes gradually increases at the onset of ripening and reaches the highest level at about 20 days before the harvest period [39]. We found that the endogenous ABA concentration in SBC berries decreased from the onset of ripening, but increased in SBBM from véraison to maturation (Fig 3). These observations agree well with those in the abovementioned studies and suggest that the concentrations of ABA lead to early ripening. Based on the physiological indices of the ABA content, gene expression led to a similar conclusion. A key enzyme in ABA biosynthesis in plants is NCED. Soar [40] found that the profile of ABA accumulation appears to roughly correlate with the expression of the *VvNCED1* gene, and Mei et al. [41] subsequently found that *VvNCED1* initiates ABA biosynthesis at the onset of fruit ripening. Also, during tomato fruit ripening, *SlNCED1* has been implicated as a key gene involved in ABA metabolism [42]. The *GlNCED1* gene is closely related to *Arabidopsis thaliana NCED6* [43], which is involved in ABA biosynthesis *in vivo* [44]. The expression of *NECD6* gene at véraison was lower than period of inflation in our experiment. This result suggest that *NCED*6 expression roughly like correlates with *NCED1*expression and is involved in ABA biosynthesis in grape, as the expression level is higher at the onset of fruit ripening, and is then down-regulated at véraison. Previous studies have shown that anthocyanin accumulation increases with colour deepened, although there is little effect of ABA on sugar accumulation [45, 46]. The effect of genes related to ABA biosynthesis in grapes has been described in some detail [47]. We have shown that the ABA level increased at the onset of ripening after decreasing at the beginning of grape maturation (Fig 3), and the qRT-PCR results (Fig 10) show that *NCED6* expression was higher at the onset of ripening. Thus, ABA might promote anthocyanin accumulation.

### *ERF*-related genes might regulate early berry ripening in SBBM

Ethylene response factor transcription factors might affect fruit ripening in climacteric fruits [48–50]. Similarly, it has been shown that ethylene is required for fruit ripening in strawberry [48]. As early as the 1970s, it was shown that ethylene levels do not increase in grape berries at véraison [49, 50]. On the contrary, a modest peak in ethylene was observed seven weeks after flowering, accompanied by a higher transcript level of 1-aminocyclopropane-1-carboxylic acid oxidase (ACO) and enzyme activity at berry colouration peaks [51]. These results support the view that at, or just prior to véraison, a modest increase in ethylene biosynthesis occurs. For example, several peaks in ethylene levels are observed in litchi at different stages of development [52], and increases in ethylene levels have also been reported in other non-climacteric fruit, e.g., strawberries [53]. *ERF5*is a transcriptional activator in *Arabidopsis*, whereas *ERF3* is an active repressor in tobacco [54]. The ERF proteins have been implicated in ethylene synthesis [55] and the expression of *ERF3* and *ERF5*correspond with ethylene production. In this study, although the expression of *ERF3* was extremely lower than *ERF5* gene, both *ERF3* and *ERF5* expression increased at the onset of ripening, and therefore, might promote early berry ripening in SBBM.

The recombinant enzymatic profiles of *Dv3MaT*are closely associated with native anthocyanin malonyltransferase activity in dahlia flowers [56], as the *Dv3MaT* transcript is abundant in red stems and red petals. Similarly, the *Dv3MaT* transcript was abundant during véraison, and was expressed at a lower level in the period of inflationin SBBM. Our results shows *3MaT*gene expressed at a lower level from period of inflation to véraison, but expressed increasing (Fig 10). Therefore, the role of *3MaT*gene might not only function in pigment stability in the flowers, but also in grape berries.

The function of *PT106* gene is probable sugar phosphate/phosphate translocator, and we have measured the soluble solids were less than 10 BX from period of inflation to period of véraison, according with the expressed of *PT106* gene showed very low level from period of inflation to period of véraison. The function of *AI5L2* gene is abscisic acid-insensitive 5-like protein, the expression of *AI5L2* gene was increased at a low level, with concentrate of ABA increasing from period of inflation to period of véraison.

### The *SEPALLATA* (*SEP*) gene probably affects grape ripening via ethylene response

The *CaMADS-RIN* gene in pepper belongs to the *SEP* clade and affects fruit ripening both in ethylene-independent and ethylene-dependent pathways in tomato [57]. Previous research has suggested that the *SlMADS-RIN* gene is bound to the *cis*-element of ACS2, which controls biosynthesis of ethylene[58]. In apple trees that showed suppressed expression of *MADS8/9*, which belongs to the *SEPALLATA1/2* (*SEP1/2*) class, severely reduced ripening and a low ethylene content were observed [59]. Seymour et al. [60] demonstrated that silencing a SEP1/2-like (*FaMADS9*) gene in strawberry led to suppressed ripening in achene and receptacle tissue. It can therefore be concluded that *SEP* genes play a key role in the regulation of ripening in both climacteric and non-climacteric fruits. In this study, compared to the locus in the reference genome, the *SEPALLATA1* gene in SBC contained a synonymous mutation, whereas S/SV/F variation was present at the locus in SBBM, in which four bases were deleted (Table 8). The expression level of *SEP1* (GSVIVT01008139001) was severely reduced when the pericarp darkened in SBBM, whereas expression of the *ERF3* and *ERF5* genes increased when the colour deepened. Therefore, we concluded that *SEP* probably down-regulated grape ripening via ethylene signaling or response.

### The anthocyanin content and *CHS* gene expression are possibly associated with early berry ripening

Anthocyanin accumulation is considered to contribute to the colouration of red and black grapes, this has been studied extensively in grapes, and anthocyanins are usually located in the pericarp of the berry. As the concentration of anthocyanins increases, the berry colour deepens. Measurements of the final anthocyanin content of the three different colours of grape varied considerably in this study: CGF contained -0.003 ng•g^-1^FW anthocyanin, whereas BMRF contained 0.06433 ng•g^-1^FWand BMPF contained 0.34333 ng•g^-1^ FW(Fig 2). During berry colouring and ripening, when the content of anthocyanins and soluble solids increased, the organic acid content decreased. The anthocyanin concentration also showed significant differences in all three types of grape.

Genes encoding phenylalanine ammonialyase (*PAL*), chalcone synthase (*CHS*), chalcone isomerase (*CHI*), flavanone 3-hydroxylase (*F3H*), dihydroflavonol 4-reductase (*DFR*), leucoanthocyanidin dioxygenase (*LDOX*), and flavonoid 3-O-glucosyltransferase (*UFGT*) and many other key genes are involved in the anthocyanin biosynthesis pathway [61]. Following isogene functional annotation and filtering, the *CHS* gene was screened and in SBBM, was found to contain a NS/SV deletion of 13,541 bases from 24,674,193 to 24,687,822 on chr14, whereas in SBC, the locus was NS compared with the reference genome. Previous research has suggested that the expression of *CHS3* correlates with anthocyanin accumulation in berry skins [62]. In *Camellia chekiangoleosa*, the expression of *CHS3* genes involved in anthocyanin biosynthesis was found, using transcriptome deep sequencing, to increase during véraison, after initially decreasing [63]. This observation agrees well with our results for grape berries.

In addition, genes encoding WD40repeat proteins regulate anthocyanin biosynthesis during fruit development in red pear [64]. The MYB–bHLH–WD40 (MBW) complex consists of different bHLH and MYB transcription factors, as well as the WD40 factor TTG1, which activates anthocyanin synthesis in vegetative tissues and *Arabidopsis* seeds [65]. The MYB114 transcription factor participates in the MBW complex, and various MBW complexes can be formed by the interaction between MYB114, and TTG1 and others, as shown by yeast two-hybrid screens [65]. S3 Table shows the transcription factor MYB114 (GSVIVT01022657001) in SBC and the transcription factor MYB114 (GSVIVT01022661001) in SBBM, which were screened and found to possess S/NS variations. Our data suggest that a large number of genes possessed S/NS/SV/F variations compared with the reference genome. Among these, genes associated with hormones, sugar, anthocyanidins, polyamine, and other factors were filtered. In addition to hormones, sugars and anthocyanidins, polyamine is also related to fruit ripening. The specific relationships among these homologous genes with fruit ripening need to be further researched.

## Conclusions

Based on whole genome re-sequence analysis, 3,692,777 SNPs and 81,223 SVs in SBC and 3,823,464 SNPs and 85,801 SVs in SBBM were screened and found to distinguish these genomes from the reference genome. We found 166 homologous unigenes related to ripening and colour in SBC and SBBM. Compared with the reference genome, the number of genes in the two lines (SBC and SBBM, respectively) that possessed NS, SVs or F was as follows: ABA (1,921 and 1,922), ETH (1,294 and 1,302), BRs (733 and 724), GAs (533 and 520), sugar (601 and 601), IAAs (1,397 and 1,405), CTKs (425 and 420), anthocyanidins (223 and 221), MYB (338 and 344), bHLH (122 and 116), WD40 (205 and 207), salicylic acid (1,504 and 1,504), and polyamine (269 and 271). The *CHS* (GSVIVT01032968001) gene was potentially related to early berry ripening and the *ERF* genes (GSVIVT01013934001 and GSVIVT01026334001) might regulate early berry ripening in SBBM. The *SEP* gene (GSVIVT01008139001) probably functions in grape ripening via ethylene response. The expression of *NCED6* (GSVIVT01029057001) appeared to roughly correlate with *NCED1* expression and might be involved in ABA biosynthesis in grape, because ABA promotes anthocyanin accumulation.

## Additional Information

Accession codes: The two raw sequencing data of this paper have been deposited at SRA under accession SRP067230, the BioProject is PRJNA304490. The data for ‘Summer Black’ grape have been deposited at BioSample under accession SAMN04299023. The data for ‘Summer Black’ bud mutation grape have been deposited at BioSample under accession SAMN04299024.

## Supporting Information

S1 Table Databases and linkage websites. (DOCX)

S2 Table The 48 pairs of primers used to confirm differences in expression between SBC and SBBM. (DOCX)

S3 Table Different uingenes associated with ripening and changes in colour in two cultivated species compared with the reference, respectively: 90 uingenes for SBC and 76 uingenes for SBBM. (DOCX)
