# Supplementary material for: Genome-Wide Detection of SNP and SV Variations to Reveal Early Ripening-Related Genes in Grape
Source: PLoS One. 2016 Feb 3;11(2):e0147749. doi: 10.1371/journal.pone.0147749 (PMC4740429; doi:10.1371/journal.pone.0147749)
Supplement: S1 Table — (DOCX) [file pone.0147749.s001.docx]

**S1 Table Databases and linkage website**

| databases Name | Linkage |
| --- | --- |
| GO  KEGG  COG  Swissprot  Nr | http://www.geneontology.org/  http://www.genome.jp/kegg/  http://www.ncbi.nlm.nih.gov/COG/  <http://www.uniprot.org/>  http://www.ncbi.nlm.nih.gov/ |
