# Supplementary material for: Genome-Wide Detection of SNP and SV Variations to Reveal Early Ripening-Related Genes in Grape
Source: PLoS One. 2016 Feb 3;11(2):e0147749. doi: 10.1371/journal.pone.0147749 (PMC4740429; doi:10.1371/journal.pone.0147749)
Supplement: S2 Table — (DOCX) [file pone.0147749.s002.docx]

**S2 Table 48 pairs of primers were used for verified the difference between SBC and SBBM**

| Accession number | Forward primer | Reverse primer |
| --- | --- | --- |
| BV140704 | TTTTCTCCCTACTCTTAACTTC | GGTAGACCTTGAAATGAAGTAA |
| BV722839 | CTCCACTTCACATCACATGGCATGC | CGGCCAACATTTACTCATCTCTCCC |
| BV140702 | AATTAATGAAGTCTGCCTAGGC | ACGAGTGAAACTTCCTAGCAAT |
| CF211871 | AGAGAGCAAAGGAACATGAA | ACAAACCCTAACCCTAGCTC |
| CV094448 | CTCTTCTCAAACTCCAATGC | AGGAGTCACCAATGATGAAG |
| BV681759 | GGGGTCCAATGTGGACTTTATC | CCATGAACAACAAACATGGCTT |
| BV722616 | TGGGGATATGCAACTTCAACTG | AATATCCCTTCAAAAATTGCCGT |
| BV722774 | ATGCCCGAGAAGAGTCGAGAA | CTGCCGTTTGGGTAAGATGCT |
| BV722695 | CTCCATCCCTATCTCATCAG | CTCTAACACCCAATCTCACA |
| BV681658 | ACCCAGCTCCTTAGCTCCTCA | GGAAGCAAAGCAGACCACAAC |
| BV681675 | GGAAGCAAAGCAGACCACAAC | CCCAGCTCCTTAGCTCCTCAC |
| BV140696 | TGCCAAAGCAAGTATCAACATG | ATTTTGATCCCACCTAACTCTG |
| BV681755 | CACAGCTGTTCCAAGTCCCA | ACAAGCCTTCCGCCACTCTC |
| G64024 | CCATCAGTGATAAAACCTAATGCC | CCCACCTTGCCCTTAGATGTTA |
| BV722854 | ACCTGGCCCGACTCCTCTTGTATGC | TCCTGCCGGCGATAACCAAGCTATG |
| BV005169 | CACGCAATCTCTCATTTCACAAA | TGGTTTAGGTGACCCAACCTTTA |
| CB977433 | GGACTTCATCCTGGAGTACA | CTTGCAGGACACCTAAATTC |
| DT009858 | ATTAACGAGGATGTGTTTGG | AAGGATCCATTTCACATACG |
| CB968692 | CCATCTACCATCACCTTTGT | GGAGAAGTGGTATTTGGTGA |
| CN007369 | TATACTTCACCGCAATTCCT | TGATCAGCTCCTCGATATTT |
| CO819364 | GGCCTCCAGATCAACTAGTAA | GCGCCTCTGTCATAGAATAC |
| DT012268 | CTCCGTGAGAGAAGGTTATG | CATTCCTGACAACCATGC |
| BV005171 | AAGAAAGTTTGCAGTTTATGGTG | AAGATGACAATAGCGAGAGAGAA |
| BV097013 | CAAACTGCCCTTCCTTTTTG | TTGCATATGCCACAAGTGATT |
| BV097000 | TGTATGATAATCCATAATGTGC | TAGGCATGCTTGACTTATTC |
| BV097005 | TGCCAATGGTTGACAAGATG | CTCGGACATGTAAGCAATCG |
| CF519163 | ATCTGACAAAGGAAAGGAGAA | GTAACATACCGAGGAAGGCA |
| BV722716 | ATTGCTTCCAAAAAGAGA | ACCCAAACCCAAATAGAT |
| BV722748 | AAGCAATGAACACAACATTCTCC | CTAAGTTTCTATGACACTTTCCTCCA |
| CV093192 | TCCAACAACAACAACTACTAC | AGGAATCTCGTGTCGCTC |
| BV102437 | CATTTCATAGGGTTTTCACAGC | CTGCCAGTATACTGATTCCTCTC |
| CN548152 | AGTCTCTTCAAGTGCCTCAG | CTGCATAGACTGACGAAACA |
| VVMD34（name） | GGTACATCAGTACTTGAAATGGTTGC | TTCTCCGTAGAAGCGTAAACAGC |
| BV096989 | CATGCGTATGTGTTAGAGAGCA | CATGGCATGTGCTTTGTTAT |
| CB915165 | AGATAATGACCGCTATGTGAA | CAACAATCCCTACCCAAAC |
| CV093018 | TCATCATCCACCACAACAC | AGTCTCTTCGCATTAGGGA |
| CF208572 | TCATCCTTTCCATACAGACC | CTCCATTGGAAGACACTCAT |
| CB005343 | CACTCTCCAACTCCAGATGT | ATGTTTCCCATATTCACAGC |
| BV097017 | ACCATCCTGCAAAAAGAAAA | CCCTCTTCCCCCTCTTTCTA |
| BV681679 | TACTTGCCCAATGGGTAATGAC | ATGGCCTCCCTACAAAAGAAAC |
| BV722766 | AGCTCGGCTAGCTGCAAAATC | ACCCTTCCCTCTTCAAAACCC |
| BV140642 | TTATCTGCTTAGGGAAAACGTA | AACACACCTTGAGAAAATAGCA |
| BV140591 | AAACAATGCTGTTAACCTGGAT | AGGGGGTGTTTAGTAATTTCAA |
| BV140701 | AAAGAAAACGAGTTTGGAAGGC | TGGGTGTTCTTGCTACTATAAT |
| BV722721 | ATCCAGAGCCATAACAGATTCA | TCACAGCTTTCTCATTACCCTT |
| CF608950 | GCTACTTCTGGGAATGTTCA | AGTCCTCATAAATTCTCAAACA |
| CF607255 | AAACCCTACCGAAGTCTCTC | AAACCCTACCGAAGTCTCTC |
| BV097008 | GGGCTCTGTTGTGCACTGTA | GGGCTCTGTTGTGCACTGTA |
