# Supplementary material for: Genome-Wide Detection of SNP and SV Variations to Reveal Early Ripening-Related Genes in Grape
Source: PLoS One. 2016 Feb 3;11(2):e0147749. doi: 10.1371/journal.pone.0147749 (PMC4740429; doi:10.1371/journal.pone.0147749)
Supplement: S3 Table — (DOCX) [file pone.0147749.s003.docx]

**S3 Table Differentuingenes which associated with ripening and coloring changes in two cultivated lines compared with reference respectively.**

| SBC | Gene ID | Gene name | Protein | variation |
| --- | --- | --- | --- | --- |
| Myb | GSVIVT01022657001 | Q9FNV8 | MY114_ARATH  Transcription factor MYB114 | S/NS |
| Ethylene | GSVIVT01029658001 | Q2V6K0 | UFOG6_FRAAN  UDP-glucose flavonoid 3-O-glucosyltransferase 6 | S/NS |
|  | GSVIVT01028823001 | Q9FLX8 | WRK27_ARATH  Probable WRKY transcription factor 27 | S/NS |
|  | GSVIVT01019113001 | Q9ZVN4 | Y1500_ARATH  Probable tyrosine-protein phosphatase At1g05000 | S/NS |
|  | GSVIVT01008601001 | Q8GY42 | NAC25_ARATH  NAC transcription factor 25 | S/NS |
|  | GSVIVT01008169001 | Q9C9B0 | U89B1_ARATH UDP-glycosyltransferase 89B1 | S/NS |
|  | GSVIVT01006778001 | O65554 | CIPK6_ARATH  CBL-interacting serine/threonine-protein kinase 6 | S/NS |
|  | GSVIVT01004571001 | Q42881 | 1A13_SOLLC 1-aminocyclopropane-1-carboxylate synthase 3 | S/NS |
|  | GSVIVT01003018001 | Q9SRS9 | CHIP_ARATH  E3 ubiquitin-protein ligase CHIP | S/NS |
| WD40 | GSVIVT01020364001 | Q38884 | EIF3I_ARATH  Eukaryotic translation initiation factor 3 subunit I | S/NS |
| Anthocyanidin | GSVIVT01023085001 | Q40288 | UFOG6_MANES Anthocyanidin 3-O-glucosyltransferase 6 | S |
| Abscisic acid | GSVIVT01037566001 | Q8RXN0 | AB11G_ARATH ABC transporter G family member 11 | S/NS |
|  | GSVIVT01036950001 | Q9LQ55 | DRP2B_ARATH Dynamin-2B OS=Arabidopsis thaliana | S/NS |
|  | GSVIVT01035777001 | O80452 | AMPD_ARATH  AMP deaminase | S/NS |
|  | GSVIVT01035679001 | Q08298 | RD22_ARATH Dehydration-responsive protein RD22 | S/NS |
|  | GSVIVT01033216001 | Q9LES3 | AI5L2_ARATH  ABSCISIC ACID-INSENSITIVE 5-like protein 2 | Sv |
|  | GSVIVT01031791001 | Q948U0 | RBOHA_SOLTU Respiratory burst oxidase homolog protein A | S/NS |
|  | GSVIVT01031731001 | K14432 | vvi:100243434\|hypothetical protein LOC100243434（KEGG） | S/NS |
|  | GSVIVT01029658001 | Q2V6K0 | UFOG6_FRAAN UDP-glucose flavonoid 3-O-glucosyltransferase 6 | S/NS |
|  | GSVIVT01027803001 | Q96303 | PHT14_ARATH  Inorganic phosphate transporter 1-4 | S/NS |
|  | GSVIVT01023401001 | Q8GUN5 | PHL1_ARATH  Protein PHR1-LIKE 1 | S/NS |
|  | GSVIVT01021351001 | Q39224 | SRG1_ARATH  Protein SRG1 | S/NS |
|  | GSVIVT01019113001 | Q9ZVN4 | Y1500_ARATH  Probable tyrosine-protein phosphatase At1g05000 | S/NS |
|  | GSVIVT01011414001 | Q9SB61 | Y4466_ARATH  ZF-HD homeobox protein At4g24660 | S/NS |
|  | GSVIVT01011225001 | CBI35227.3 | 296087944 | S/Sv |
|  | GSVIVT01008601001 | Q8GY42 | NAC25_ARATH  NAC transcription factor 25 | S/NS |
|  | GSVIVT01008169001 | Q9C9B0 | U89B1_ARATH UDP-glycosyltransferase 89B1 | S/NS |
|  | GSVIVT01007133001 | CBI23529.3 | 297735952 | S/NS |
|  | GSVIVT01006778001 | O65554 | CIPK6_ARATH  CBL-interacting serine/threonine-protein kinase 6 | S/NS |
|  | GSVIVT01005410001 | Q8H1H9 | AMO_ARATH  Primary amine oxidase | Sv/F |
|  | GSVIVT01005140001 | Q94B74 | NUDT2_ARATH Nudix hydrolase 2 | S/NS |
|  | GSVIVT01003018001 | Q9SRS9 | CHIP_ARATH  E3 ubiquitin-protein ligase CHIP | S/NS |
|  | GSVIVT01002663001 | Q8RXN0 | AB11G_ARATH  ABC transporter G family member 11 | S/NS |
|  | GSVIVT01002361001 | P49315 | CATA1_NICPL  Catalase isozyme 1 | S/NS |
| Cytokinin | GSVIVT01023401001 | Q8GUN5 | PHL1_ARATH  Protein PHR1-LIKE 1 | S/NS |
|  | GSVIVT01013497001 | O80366 | ARR9_ARATH Two-component response regulator ARR9 | S/NS |
|  | GSVIVT01011414001 | Q9SB61 | Y4466_ARATH  ZF-HD homeobox protein At4g24660 | S/NS |
|  | GSVIVT01004571001 | Q42881 | 1A13_SOLLC 1-aminocyclopropane-1-carboxylate synthase 3 | S/NS |
| Gibberellin | GSVIVT01033216001 | Q9LES3 | AI5L2_ARATH  ABSCISIC ACID-INSENSITIVE 5-like protein 2 | Sv |
|  | GSVIVT01032935001 | Q9FL79 | EXP23_ARATH Expansin-A23 | Sv |
|  | GSVIVT01018453001 | O04705 | GAO1D_WHEAT  Gibberellin 20 oxidase 1-D | S/NS |
|  | GSVIVT01011414001 | Q9SB61 | Y4466_ARATH  ZF-HD homeobox protein At4g24660 | S/NS |
|  | GSVIVT01007987001 | Q9C554 | EXPA1_ARATH Expansin-A1 | S/NS |
|  | GSVIVT01007798001 | Q9FJ93 | DRE1D_ARATH Dehydration-responsive element-binding protein 1D | S/NS |
| Sugar | GSVIVT01035777001 | O80452 | AMPD_ARATH  AMP deaminase | S/NS |
|  | GSVIVT01028713001 | Q6NQN5 | SWET3_ARATH Bidirectional sugar transporter SWEET3 | S/NS |
|  | GSVIVT01027803001 | Q96303 | PHT14_ARATH  Inorganic phosphate transporter 1-4 | S/NS |
|  | GSVIVT01009047001 | Q9LIQ7 | PP252_ARATH Pentatricopeptide repeat-containing protein At3g24000, mitochondrial | Sv |
|  | GSVIVT01007765001 | Q8GYT9 | SIS3_ARATH  E3 ubiquitin-protein ligase SIS3 | S/NS |
| bHLH | GSVIVT01008093001 | Q9LV17 | BH079_ARATH Transcription factor bHLH79 | S/NS |
| Polyamine | GSVIVT01022657001 | Q9FNV8 | MY114_ARATH Transcription factor MYB114 | S/NS |
|  | GSVIVT01009793001 | Q9SCV9 | BGAL3_ARATH Beta-galactosidase 3 | S/NS |
| Auxin | GSVIVT01036417001 | B9JEB9 | NQOR_AGRRK  NAD(P)H dehydrogenase （quinone） | S/NS |
|  | GSVIVT01033354001 | O82333 | GH31_ARATH  Probable indole-3-acetic acid-amidosynthetase GH3.1 | Sv |
|  | GSVIVT01031791001 | Q948U0 | RBOHA_SOLTU Respiratory burst oxidase homolog protein A | S/NS |
|  | GSVIVT01028295001 | Q9SLB7 | LBD16_ARATH  LOB domain-containing protein 16 | S/NS |
|  | GSVIVT01027161001 | P24465 | C71A1_PERAE Cytochrome P450 71A1 | S/NS |
|  | GSVIVT01026220001 | A5AEM3 | THS4_VITVI  Stilbene synthase 4 | S/NS |
|  | GSVIVT01021809001 | C6TBN2 | AKR1_SOYBN  Probable aldo-keto reductase 1 | S/NS |
|  | GSVIVT01018453001 | O04705 | GAO1D_WHEAT  Gibberellin 20 oxidase 1-D | S/NS |
|  | GSVIVT01014948001 | Q05047 | C72A1_CATRO Secologanin synthase | S/NS |
|  | GSVIVT01011414001 | Q9SB61 | Y4466_ARATH  ZF-HD homeobox protein At4g24660 | S/NS |
|  | GSVIVT01008601001 | Q8GY42 | NAC25_ARATH  NAC transcription factor 25 | S/NS |
|  | GSVIVT01008109001 | Q9FME3 | TCP5_ARATH Transcription factor TCP5 | S/NS |
|  | GSVIVT01004571001 | Q42881 | 1A13_SOLLC 1-aminocyclopropane-1-carboxylate synthase 3 | S/NS |
|  | GSVIVT01003018001 | Q9SRS9 | CHIP_ARATH  E3 ubiquitin-protein ligase CHIP | S/NS |
| Brassinosteroid | GSVIVT01033354001 | O82333 | GH31_ARATH  Probable indole-3-acetic acid-amidosynthetase GH3.1 | S/NS |
|  | GSVIVT01028295001 | Q9SLB7 | LBD16_ARATH  LOB domain-containing protein 16 | S/NS |
|  | GSVIVT01025771001 | Q93Y09 | SCP45_ARATH  Serine carboxypeptidase-like 45 | S/NS |
|  | GSVIVT01025312001 | Q00874 | DR100_ARATH DNA-damage-repair/toleration protein DRT100 | S/NS |
|  | GSVIVT01015445001 | Q93Z92 | RING4_ARATH  E3 ubiquitin-protein ligase At4g11680 | S/NS |
|  | GSVIVT01014948001 | Q05047 | C72A1_CATRO Secologanin synthase | S/NS |
|  | GSVIVT01011816001 | Q9FID5 | Y5393_ARATH  Probable receptor-like protein kinase At5g39030 | S/NS |
|  | GSVIVT01011414001 | Q9SB61 | Y4466_ARATH  ZF-HD homeobox protein At4g24660 | S/NS |
|  | GSVIVT01009793001 | Q9SCV9 | BGAL3_ARATH Beta-galactosidase 3 | S/NS |
|  | GSVIVT01009517001 | Q9FMZ0 | BKI1_ARATH  BRI1 kinase inhibitor 1 | S/NS |
|  | GSVIVT01004774001 | B0UV30 | TAL_HAES2  Transaldolase | S/NS |
| Salicylic acid | GSVIVT01034031001 | Q9M2S4 | LRKS4_ARATH  L-type lectin-domain containing receptor kinase S.4 | S/NS |
|  | GSVIVT01031791001 | Q948U0 | RBOHA_SOLTU Respiratory burst oxidase homolog protein A | S/NS |
|  | GSVIVT01029316001 | P93484 | VSR1_PEA  Vacuolar-sorting receptor 1 | S/NS |
|  | GSVIVT01027147001 | Q39828 | SDL5A_SOYBN Dynamin-related protein 5A | S/NS |
|  | GSVIVT01026685001 | F4I171 | MD15A_ARATH  Mediator of RNA polymerase II transcription subunit 15a | S/NS |
|  | GSVIVT01026677001 | Q9LK64 | AB3C_ARATH  ABC transporter C family member 3 | S/NS |
|  | GSVIVT01024306001 | O49835 | PAL1_LITER  Phenylalanine ammonia-lyase 1 | S/NS |
|  | GSVIVT01019209001 | P32110 | GSTX6_SOYBN  Probable glutathione S-transferase | S/NS |
|  | GSVIVT01016941001 | C0LGD6 | Y1570_ARATH  Probable LRR receptor-like serine/threonine-protein kinase At1g05700 | S/NS |
|  | GSVIVT01011816001 | Q9FID5 | Y5393_ARATH  Probable receptor-like protein kinase At5g39030 | S/NS |
|  | GSVIVT01008036001 | P43293 | NAK_ARATH Probable serine/threonine-protein kinase NAK | S/NS |
|  | GSVIVT01005706001 | O81905 | SD18_ARATH  Receptor-like serine/threonine-protein kinase | S/NS |
|  | GSVIVT01005140001 | Q94B74 | NUDT2_ARATH  Nudix hydrolase 2 | S/NS |

| SBBM | Gene ID | Gene name | Protein name | variation |
| --- | --- | --- | --- | --- |
| Myb | GSVIVT01022661001 | Q9FNV8 | MY114_ARATH  Transcription factor MYB114 | S/NS |
|  | GSVIVT01011986001 | Q9S7G7 | MB3R1_ARATH  Myb-related protein 3R-1 | S/NS |
|  | GSVIVT01008005001 | P81392 | MYB06_ANTMA  Myb-related protein 306 | S/NS |
| Ethylene | GSVIVT01034455001 | P52835 | F3ST_FLABI  Flavonol 3-sulfotransferase | S/NS |
|  | GSVIVT01026334001 | Q94AW5 | ERF03_ARATH  Ethylene-responsive transcription factor ERF003 | Sv |
|  | GSVIVT01008146001 | Q9ZPQ5 | SOT13_ARATH  Cytosolic sulfotransferase 13 | S/NS |
|  | GSVIVT01004272001 | Q651X6 | CSLE6_ORYSJ  Cellulose synthase-like protein E6 | S/NS |
|  | GSVIVT01003682001 | Q651X6 | SD113_ARATH  G-type lectin S-receptor-like serine/threonine-protein kinase SD1-13 | S/NS |
|  | GSVIVT01002536001 | Q52QU2 | AIL6_ARATH  AP2-like ethylene-responsive transcription factor AIL6 | S/NS |
|  | GSVIVT01003084001 | F4JKI3 | DGK6_ARATH  Diacylglycerol kinase 6 | S/NS |
| WD40 | GSVIVT01008513001 | Q9M3B4 | WD40 repeat [R]  RID3_ARATH  Protein ROOT INITIATION DEFECTIVE | S/NS |
|  | GSVIVT01007685001 | Q5ZME8 | SMU1_CHICK  WD40 repeat-containing protein SMU1 | S |
| Anthocyanidin | GSVIVT01013693001 | Q5ZCW1 | LAC1_ORYSJ  Putative laccase-1 | S |
| Abscisic acid | GSVIVT01036492001 | P31169 | KIN2_ARATH  Stress-induced protein KIN2 | S |
|  | GSVIVT01034532001 | Q50EK0 | C16B2_PICSI  Cytochrome P450 | S/NS |
|  | GSVIVT01034455001 | P52835 | F3ST_FLABI  Flavonol 3-sulfotransferase | S/NS |
|  | GSVIVT01034321001 | B9DHT4 | ARIA_ARATH  ARM REPEAT PROTEIN INTERACTING WITH ABF2 | S/NS |
|  | GSVIVT01034321001 | B9DHT4 | ARIA_ARATH  ARM REPEAT PROTEIN INTERACTING WITH ABF2stimulus (GO:0009737) | S/NS |
|  | GSVIVT01031284001 | C7G304 | GOLS2_SOLLC Galactinol synthase 2 | S/NS |
|  | GSVIVT01030973001 | Q8LFD1 | LPP3_ARATH  Putative lipid phosphate phosphatase 3, chloroplastic | S/NS |
|  | GSVIVT01029406001 | Q8H191 | PAO4_ARATH  Probable polyamine oxidase 4 | S/NS |
|  | GSVIVT01027372001 | Q54YW1 | ELMOA_DICDIELMO  domain-containing protein A | S/NS |
|  | GSVIVT01026976001 | P46519\| | LEA14_SOYBN  Desiccation protectant protein Lea14 homolog | S/NS |
|  | GSVIVT01026905001 | P37116 | NCPR_VIGRR  NADPH--cytochrome P450 reductase | S/NS |
|  | GSVIVT01026334001 | Q94AW5 | \|ERF03_ARATH  Ethylene-responsive transcription factor ERF003 | Sv |
|  | GSVIVT01026323001 | O04161 | AMT12_SOLLC  Ammonium transporter 1 member 2 | S/NS |
|  | GSVIVT01021334001 | Q39224 | SRG1_ARATH  Protein SRG1 | S/NS |
|  | GSVIVT01018937001 | Q9ZPV9 | SY112_ARATH  Syntaxin-112 | Sv |
|  | GSVIVT01011098001 | Q93ZT5 | EDL3_ARATH  EID1-like F-box protein 3 | S/NS |
|  | GSVIVT01008069001 | Q94K41 | SDR3B_ARATH  Short-chain dehydrogenase reductase 3b | S/NS |
|  | GSVIVT01008005001 | P81392 | MYB06_ANTMA  Myb-related protein 306 | S/NS |
|  | GSVIVT01002864001 | Q8H1D6 | RBK1_ARATH  Receptor-like cytosolic serine/threonine-protein kinase RBK1 | S/NS |
|  | GSVIVT01002536001 | Q52QU2 | AIL6_ARATH  AP2-like ethylene-responsive transcription factor AIL6 | S/NS |
|  | GSVIVT01002319001 | Q8RXN0 | AB11G_ARATH  ABC transporter G family member | S/NS |
|  | GSVIVT01002139001 | Q8RXN0 | AB11G_ARATH  ABC transporter G family member 11 | S/NS |
|  | GSVIVT01002136001 | Q8RXN0 | AB11G_ARATH  ABC transporter G family member 11 | S/NS |
| Cytokinin | GSVIVT01034455001 | P52835 | F3ST_FLABI  Flavonol 3-sulfotransferase | S/NS |
|  | GSVIVT01026334001 | Q94AW5 | ERF03_ARATH  Ethylene-responsive transcription factor ERF003 | Sv |
|  | GSVIVT01008146001 | Q9ZPQ5 | SOT13_ARATH  Cytosolic sulfotransferase 13 | S/NS |
| Gibberellin | GSVIVT01031827001 | Q9C899 | F6H2_ARATH  Feruloyl CoA ortho-hydroxylase 2 | S/NS |
|  | GSVIVT01030192001 | Q40636 | EXPA2_ORYSJ  Expansin-A2 | S/NS |
|  | GSVIVT01008005001 | P81392 | MYB06_ANTMA  Myb-related protein 306 | S/NS |
| Sugar | GSVIVT01002623001 | Q9C5H6 | CSTR3_ARATH  CMP-sialic acid transporter 3 | S/NS |
| bHLH | GSVIVT01037927001 | Q9SA82 | BH052_ARATH  Transcription factor bHLH52 | S/NS |
| Polyamine | GSVIVT01029406001 | Q8H191 | PAO4_ARATH  Probable polyamine oxidase 4 | S/NS |
|  | GSVIVT01022661001 | Q9FNV8 | MY114_ARATH  Transcription factor MYB114 | S/NS |
|  | GSVIVT01008005001 | P81392 | MYB06_ANTMA  Myb-related protein 306 | S/NS |
|  | GSVIVT01001759001 | CBI38305.3\| | 296088847(NR) | S/NS |
| Auxin | GSVIVT01036750001 | Q39182 | DEF02_ARATH  Defensin-like protein 2 | S/NS |
|  | GSVIVT01034455001 | P52835 | F3ST_FLABI  Flavonol 3-sulfotransferase | S/NS |
|  | GSVIVT01031230001 | P31414 | AVP1_ARATH  Pyrophosphate-energized vacuolar membrane proton pump 1 | S/NS |
|  | GSVIVT01029176001 | P35694 | BRU1_SOYBN  Brassinosteroid-regulated protein BRU1 | Sv/F |
|  | GSVIVT01024130001 | P33079 | A10A5_SOYBN  Auxin-induced protein | S/NS |
|  | GSVIVT01022710001 | O81635 | ATK4_ARATH  Kinesin-4 | S/NS |
|  | GSVIVT01017554001 | CBI36474.3 | 297743607（NR） | S/NS |
|  | GSVIVT01010274001 | Q40901 | DEF_PETIN  Defensin-like protein | S/NS |
|  | GSVIVT01008910001 | Q9ZWQ9 | FLS_CITUN  Flavonol synthase/flavanone 3-hydroxylase | S/NS |
|  | GSVIVT01008005001 | P81392 | MYB06_ANTMA  Myb-related protein 306 | S/NS |
|  | GSVIVT01007417001 | Q9SD85 | F3PH_ARATH  Flavonoid 3&apos;-monooxygenase | S/NS |
|  | GSVIVT01001248001 | Q9LTT9 | VCR_ARATH  Varicose-related protein | S/NS |
| Brassinosteroid | GSVIVT01034492001 | Q4ULG1 | ODP2_RICFE  Dihydrolipoyllysine-residue acetyltransferase component of pyruvate dehydrogenase complex | S/NS |
|  | GSVIVT01034455001 | P52835 | F3ST_FLABI  Flavonol 3-sulfotransferase | S/NS |
|  | GSVIVT01029176001 | P35694 | BRU1_SOYBN  Brassinosteroid-regulated protein BRU1 | Sv/F |
|  | GSVIVT01026334001 | Q94AW5 | ERF03_ARATH  Ethylene-responsive transcription factor ERF003 | Sv |
|  | GSVIVT01014738001 | P49351 | FPPS1_LUPAL  Farnesyl pyrophosphate synthase 1 | S/NS |
| Salicylic acid | GSVIVT01026334001 | Q94AW5 | ERF03_ARATH  Ethylene-responsive transcription factor ERF003 | Sv |
|  | GSVIVT01026323001 | O04161 | AMT12_SOLLC  Ammonium transporter 1 member 2 | S/NS |
|  | GSVIVT01024299001 | Q9SMK9 | PAL2_CICAR  Phenylalanine ammonia-lyase 2 | S/NS |
|  | GSVIVT01020775001 | Q8RWZ5 | SD25_ARATH  G-type lectin S-receptor-like serine/threonine-protein kinase SD2-5 | S/NS |
|  | GSVIVT01016947001 | Q9LIG2 | RLK6_ARATH  Receptor-like protein kinase At3g21340 | S/NS |
|  | GSVIVT01015649001 | Q9FUY6 | JOIN_SOLLC  MADS-box protein JOINTLESS | S/NS |
|  | GSVIVT01010942001 | Q9LZI9 | GSTFD_ARATH  Glutathione S-transferase F13 | S/NS |
|  | GSVIVT01008723001 | Q9SGS2 | HMGB9_ARATH  High mobility group B protein 9 | S/NS |
|  | GSVIVT01007625001 | Q9M8Y5 | LTL1_ARATH  GDSL esterase/lipase LTL1 | S/NS |
|  | GSVIVT01004272001 | Q651X6 | CSLE6_ORYSJ  Cellulose synthase-like protein E6 | S/NS |
|  | GSVIVT01003577001 | F4I171 | MD15A_ARATH  Mediator of RNA polymerase II transcription subunit 15a | S/NS |
|  | GSVIVT01003532001 | F4I171 | MD15A_ARATH  Mediator of RNA polymerase II transcription subunit 15a | S/NS |

13 key factors changes (NS, Sv, F) associated with ripening and coloring.90 uingenes for SBC and 76 uingenes for SBBM.
